# Challenges and Perspectives of DNA Nanostructures in Biomedicine

**DOI:** 10.1002/anie.201916390

**Published:** 2020-06-03

**Authors:** Adrian Keller, Veikko Linko

**Affiliations:** ^1^ Technical and Macromolecular Chemistry Paderborn University Warburger Strasse 100 33098 Paderborn Germany; ^2^ Biohybrid Materials Department of Bioproducts and Biosystems Aalto University P. O. Box 16100 00076 Aalto Finland; ^3^ HYBER Centre Department of Applied Physics Aalto University P. O. Box 15100 00076 Aalto Finland

**Keywords:** biocompatibility, diagnostics, DNA nanotechnology, drug delivery, nanomedicine

## Abstract

DNA nanotechnology holds substantial promise for future biomedical engineering and the development of novel therapies and diagnostic assays. The subnanometer‐level addressability of DNA nanostructures allows for their precise and tailored modification with numerous chemical and biological entities, which makes them fit to serve as accurate diagnostic tools and multifunctional carriers for targeted drug delivery. The absolute control over shape, size, and function enables the fabrication of tailored and dynamic devices, such as DNA nanorobots that can execute programmed tasks and react to various external stimuli. Even though several studies have demonstrated the successful operation of various biomedical DNA nanostructures both in vitro and in vivo, major obstacles remain on the path to real‐world applications of DNA‐based nanomedicine. Here, we summarize the current status of the field and the main implementations of biomedical DNA nanostructures. In particular, we focus on open challenges and untackled issues and discuss possible solutions.

## Graphical abstract

**Although DNA nanotechnology** has spawned a broad variety of biomedical nanostructures over the last decade, these DNA nanostructures often suffer from limited stability and considerable immunogenicity. This Review introduces various biomedical applications of DNA nanostructures, describes the challenges imposed on the DNA nanostructures by the physiological environment, and discusses possible solutions to these challenges.

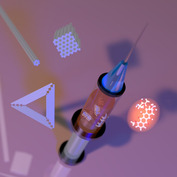

## 1 Introduction

DNA is the carrier of hereditary information and therefore serves as a central component of all life on Earth. That said, due to its unique chemical and structural properties, DNA can also be used as a programmable material^1^ for the controlled synthesis of molecularly defined artificial nanostructures and even switchable nanodevices.^2^ Long considered an exotic niche topic, the field of DNA nanotechnology has immensely grown over the past decade, both on the experimental and computational side.^3^ As an indication of this, DNA nanostructures (DN) are currently applied in research areas as diverse as nanoelectronics, chemical sensing, molecular computing, and biomedicine.^4^ The latter field in particular has recently made impressive progress toward the utilization of DN in various therapeutic and diagnostic applications.

Compared to other more conventional nanomaterials, DN have some significant advantages when it comes to biomedical applications. First, whereas many nanoparticle systems have raised concerns regarding possible adverse effects,^5^ DN are essentially biocompatible, biodegradable, and non‐cytotoxic. Second, DN and especially DNA origami (DO)^6^ can be assembled in well‐defined yet almost arbitrary sizes and shapes and thereby provide a means to tuning their biological availability and activity. Third, their surfaces can be modified in a precisely controlled manner with molecular accuracy. This is particularly true for DO, which are based on the folding of a long, single‐stranded DNA scaffold into a desired nanoscale shape by hybridization with a set of short staple strands. Each of these staples has a unique sequence and can thus be unambiguously addressed and modified to carry different entities such as dye molecules, proteins, nanoparticles, and drugs. In this way, dozens of different functional species can be arranged with nanometer accuracy on the interior and exterior surfaces of DO. This approach thus holds great promise for various biomedical applications, as it does not only enable the defined loading of the DN with various therapeutic cargos, but may also be used to facilitate cell targeting, cellular uptake, target binding, and their conformational switching in response to various external stimuli. In general, DN may bridge biochemically relevant length scales and sub‐nanometer precision to macroscopic dimensions.

However, DN are intrinsically less stable than inorganic nanomaterials, which may result in serious limitations regarding their applicability in physiological environments that are equipped with sophisticated machinery for identifying and degrading foreign DNA. Stabilizing DN under such adverse conditions without impairing their desired biomedical function thus represents the most prominent challenge that we currently face on the road to real‐world applications. In this Review, we shall first introduce the various therapeutic and diagnostic applications of DN before summarizing the challenges imposed on DN stability and functionality by the physiological environment. Finally, we will discuss strategies and solutions to these challenges and, in particular, address shortcomings, unsolved issues, and potential conflicts with regard to DN functionality.

## 2 Biomedical Applications of DNA Nanostructures

The biomedical applications of DN are just as numerous as their shapes. With some exceptions such as the field of drug discovery, which is seeing more and more DNA‐nanotechnology‐related works,^7^ most of these applications require the exposure of the DN to biological media, either in vivo or ex vivo. The latter automatically provides a distinction between the two major application areas: therapeutic applications typically aim at employing the DN inside the human body, whereas diagnostic applications often (although not always, see Section 2.2) only require exposure to (sometimes diluted or purified) blood, serum, or tissue samples. In this section, we will provide an overview of these two major application areas of DN in the biomedical field. For further in‐depth discussions of the specific applications, the reader is referred to the large number of recent Reviews that focus specifically on these topics.^8^, ^9^

### 2.1 Therapeutic Applications

Doxorubicin (DOX) is widely and commercially used in cancer therapy—especially for solid tumors—and it is a well‐known DNA intercalator by its nature. Therefore, at least in principle, it should be one of the most promising candidates for DN‐based delivery, as it can be loaded into customized nanostructures that may have a plethora of other functions. There are multiple examples of DN that have been employed as DOX carriers such as DNA tetrahedra (DT),^10^, ^11^ twisted 3D DO,^12^ DO triangles,^13^, ^14^, ^15^ rectangles,^11^ and helix bundles,^13^ as well as tubular DO loaded into liposomes.^16^ In addition to DOX, its close molecular relative daunorubicin has also been used as a drug for a (Trojan) “DNA horse”.^17^ Although the efficacy of DOX‐loaded DNA nanocarriers was verified in several in‐vivo models,^10^, ^11^, ^13^, ^14^, ^16^ each approach has its own and different loading and purification strategy, environment, pH, as well as DOX and ion concentrations, thus making the results extremely hard to compare to each other. Not only are the spectroscopic^18^ properties of DOX strongly ion‐ and pH‐dependent,^19^ but DOX is also commonly employed in substantial excess to DN in the loading process, although it is known to self‐aggregate at high concentrations.^20^ Moreover, DOX can also bind to partially hybridized or self‐hybridized staples that are used in excess to DO‐scaffold strand during folding. As the binding affinity of DOX is only slightly DNA‐sequence‐dependent,^21^ the effects of staples should not be ignored. That being the case, studies relying purely on the spectroscopic properties of DOX and not taking into account all the above‐mentioned factors may produce ambiguous results and leave plenty of room for speculation.

Besides broadly employed DOX, there are other potential drugs that can be loaded into DN. Again, the nature of the interaction between the chosen drug and DN depends on the prevalent conditions, but the loading efficiency may also depend on the DN superstructure. This has been demonstrated for intercalating YOYO‐1 and acridine orange molecules^22^ as well as for groove‐binding methylene blue.^23^

In contrast to the supposedly simple loading of the DNA nanocarriers with intercalators or groove‐binders, DN can also be employed for the spatially controlled presentation of functional molecules. Möser et al. recently conjugated ephrin‐mimicking peptides that bind to EphrinA2 receptors to the tips of a DNA three‐arm junction.^24^ Ephrin‐signaling pathways are involved in tumor development and may thus be utilized in cancer therapy. The authors observed that the oligovalent presentation of three ephrin‐mimicking peptides on one DN resulted in significantly increased EphA2 phosphorylation in PC‐3 cells compared to monomeric peptides. DN‐templated oligovalence thus represents a promising concept for various therapeutic applications. Furthermore, it was found that even monomeric peptides showed higher potency when coupled to the DN, which may provide a rather simple route to tuning drug stability, distribution, and activity.^24^

**Figure 1:**
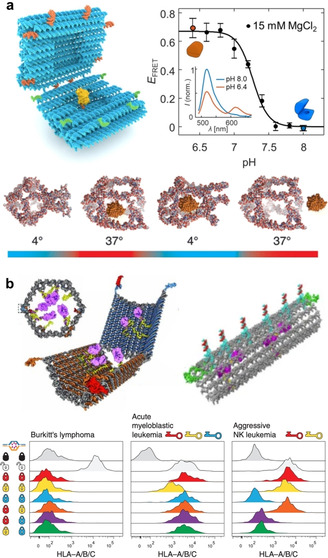
Dynamic devices for therapeutics. a) DNA nanocarriers for enzyme encapsulation and display. Top panel: A pH‐switchable DO nanocapsule that can be reversibly closed and opened. Closing and opening is characterized using Förster‐resonance energy transfer (FRET). Bottom panel: A temperature‐responsive DNA cage for enzyme trapping and release. b) DO nanorobots. Top left panel: An antibody‐loaded logic‐gated shell‐like robot. Bottom panel: Different lock and key combinations of logic‐gated robots analyzed by flow cytometry. Top right panel: A thrombin‐loaded tubular nanorobot that opens through interaction between nucleolin proteins and “fastener” strands. a) Top panel reproduced with permission from ref. 31 (https://pubs.acs.org/doi/10.1021/acsnano.9b01857). Copyright 2019 American Chemical Society. Further permissions related to the material excerpted should be directed to the American Chemical Society. Bottom panel reproduced with permission from ref. 33. Copyright 2013 American Chemical Society. b) Top left panel and bottom panel reproduced with permission from ref. 41. Copyright 2012 AAAS. Top right panel reproduced with permission from ref. 40 Copyright 2018 Springer Nature.

In cells, compartmentalization and precise organization of active compounds such as enzymes is vital for specificity, control, and enhancement of reactions. There are many routes to achieve artificial compartments for enzymes, but the unprecedented addressability of DN makes them highly attractive candidates for this purpose. Protein encapsulation is beneficial not only for multipurpose delivery applications ranging from infectious to genetic‐disease treatments,^25^ but also for modulating protein properties such as stability and function.^26^ It has been shown that the cellular delivery of luciferase‐loaded DO can be achieved and that the enzymes retain their activity in the process.^27^ Moreover, the activity of these enzymes can be further modulated with cationic polymer coatings of the hollow DO container (see also Section 4.2).^28^ There is a wide variety of DNA‐based vessels for enzymatic cargos such as cascade nanoreactors,^29^ tubular hosts,^30^ reconfigurable vaults and capsules (Figure 1 a, top panel),^31^, ^32^ and various DNA cages (Figure 1 a, bottom panel)^33^, ^34^ as well as nanosheets for nuclease delivery.^35^ These types of (multi‐)enzyme systems and vehicles with enzymatic payloads have been reviewed in Refs. 26, 36. It is noteworthy that harnessing DNA templates for protein assembly may have rather intriguing and unconventional implementations in tailored protein design, as demonstrated by Rosier et al.^37^ They assembled a functional apoptosome by co‐localization of multiple caspase‐9 monomers with the help of a DO platform. This approach may pave the way for the engineering of artificial enzymes that are involved in processes such as inflammation, innate immunity, and necrosis.

In addition to drug and enzyme delivery, DN are investigated as delivery vehicles for therapeutic nucleic acids such as small interfering RNA (siRNA), antisense RNAs (asRNA), and genes.^38^, ^39^, ^40^ In a seminal study by Lee et al., the authors employed DT to deliver siRNA sequences into cells.^39^ They observed the suppressed expression of the targeted genes only when the amount of cancer‐targeting ligands (folates) and their orientation was appropriate, thus underlining the importance of the spatial addressability of the DN. Moreover, these particles exhibited longer blood circulation half‐lives than the parental siRNA. Similar DNA shapes have also been used in a nanogel‐based siRNA‐delivery system.^40^

Very recently, Li et al. showed that aptamer‐equipped DN could be used in the repair of cerebral ischemia‐reperfusion injury (IRI) in rats.^42^ Oxidative stress combined with inflammation is the main contributor to brain IRI, which is intensified by the complement component 5a (C5a). Therefore, the authors utilized DNA frameworks conjugated with anti‐C5a aptamers to selectively reduce C5a‐mediated neurotoxicity and effectively relieve oxidative stress in the brain.

Immunostimulatory CpG oligonucleotides are a special class of therapeutic nucleic acids. These CpG sequences stimulate intracellular Toll‐like receptors in macrophages and dendritic cells, which results in T‐cell activation.^9^ They can thus be used in cancer immunotherapy and as vaccine adjuvants. Numerous studies used DN to display CpG oligonucleotides and deliver them into macrophage‐like cells without the need for transfecting agents.^43^, ^44^ Enhanced immunostimulation due to DN‐mediated CpG delivery was also validated in vivo.^45^

In contrast to chemo‐, immuno‐, or gene therapy, photodynamic^46^ and photothermal therapy^47^ employ inert compounds and materials that become active only upon interaction with light. These can be either photosensitizers that generate reactive oxygen species upon illumination or plasmonic nanomaterials such as gold nanoparticles that heat up due to the resonant absorption of photons of a certain wavelength. Many of these photosensitizer and nanoparticle systems, however, suffer from low solubility and low cell/tissue uptake. At several instances, the application of DN‐based carriers was shown to overcome these drawbacks and result in improved anticancer activity both in vitro and in vivo.^48^, ^49^, ^50^

On top of the above‐mentioned examples, DN addressability, activity, and emerging multifunctionality may find uses in targeted therapy, where the designed vehicle can perform multiple tasks. One of the earliest works manifesting this kind of utility was a dynamic logic‐gated nanorobot designed by Douglas et al. (Figure 1 b, top left panel).^41^ The authors equipped a hollow‐shell‐like and spring‐loaded DO with antibodies and closed the device using an aptamer “lock” system (dsDNA) that can only be opened through binding of a specific antigen “key”. They demonstrated the logic gating with a two‐input system by assembling a logic AND gate through aptamer encoding, that is, a gate where both locks needed to be opened simultaneously to activate the robot (Figure 1 b, bottom panel). Furthermore, they presented the versatility of the system by building a handful of distinct versions of these aptamer‐encoded devices and testing the response with multiple different cell lines. Later on, similar nanorobots were employed in performing universal computing in living cockroaches.^51^ This approach by Amir et al. was based on dynamic interactions between the robots. These interactions served as logical outputs that were further relayed to switch molecular payloads on or off, thus opening new avenues in the computational control of therapeutics.

Along these lines, Li et al. created their own version of a dynamic in‐vivo nanorobot (Figure 1 b, top right panel).^52^ They used a thrombin‐loaded rectangular DO that was further wrapped into a tubular shape with DNA “fastener” strands and functionalized with targeting aptamers. The fastener strands were designed in such a way they could open through the interaction with nucleolin proteins that are expressed at the surface of actively proliferating tumor vascular endothelial cells. When the encapsulated cargo was displayed and exposed through the nucleolin‐induced reconfiguration of the robot, thrombin activated blood coagulation at the tumor site. The authors used mice to demonstrate the specific delivery of robots to tumor‐associated blood vessels and the resulting intravascular thrombosis. Finally, this led to tumor necrosis and inhibition of tumor growth.

Related to above examples, Liu et al. have also shown that drug‐molecule‐loaded delivery devices with multifunctional properties can be assembled using the DO technique.^14^ The authors integrated gene delivery with cancer therapy by loading a DO triangle with DOX molecules and equipping it with two linear tumor‐therapeutic genes (p53). In a similar manner, the same group used a combination of RNA interference (RNAi) and chemotherapy by incorporating siRNA and DOX into a single DO.^53^ These multifunctional devices could enter multidrug‐resistant tumors (MCF‐7R) and inhibit their growth both in vitro and in vivo. Furthermore, the research group of Ding has also shown that DOX, gold nanorods, and the tumor‐specific aptamer MUC‐1 can all be incorporated into one DO vehicle for effective circumvention of drug resistance.^54^

**Figure 2:**
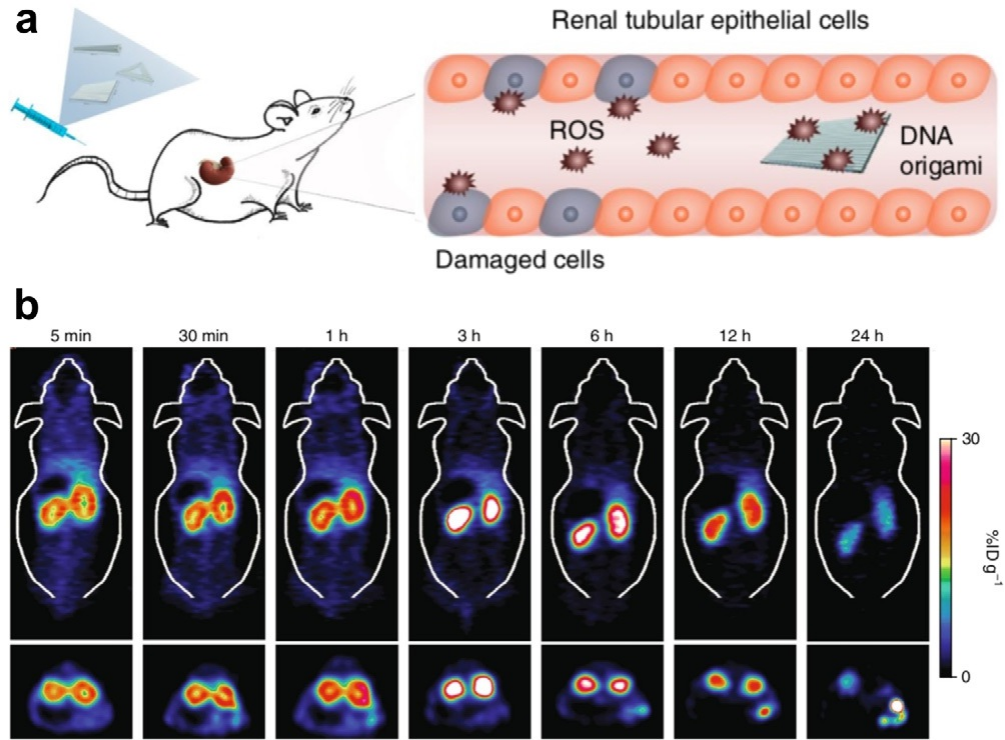
DN against acute kidney injury (AKI). a) Schematics of using non‐modified DO as therapeutics in mice: Rectangular DO alleviate AKI via scavenging of reactive oxygen species (ROS). b) Positron‐emission tomography (PET) shows rapid accumulation of ^64^Cu‐labeled DO in the kidneys of mice with AKI. Reproduced with permission from ref. 55. Copyright 2018 Springer Nature.

Jiang et al. recently demonstrated that even non‐modified DO can have therapeutic potential (Figure 2).^55^ They observed that the DO preferentially ended up in the kidneys (Figure 2 b), whereas partially folded nanostructures and unfolded scaffold strands were sequestered by the liver or experienced rapid renal clearance. Most astonishingly, the authors also found that at least one of the DO shapes exhibited renal‐protective properties. Rectangular DO could efficiently alleviate acute kidney injury (AKI) in mice via the scavenging of reactive oxygen species (ROS, Figure 2 a). The therapeutic response was rapid, which indicates that DO may be promising candidates for the treatment of various kidney‐related diseases. Importantly, the authors also showed that the selected rectangular DO shape was not toxic to organ functions and it did not elicit an immune response in vivo. However, it should be pointed out that DO may still require additional protection mechanisms for efficient targeting and enhanced pharmacokinetic bioavailability, which might change their biodistribution from what has been shown here. Nevertheless, the possibility of using DN themselves as drugs by exploiting their ROS‐scavenging abilities is very appealing and deserves further in‐depth investigation.

### 2.2 Diagnostic Applications

**Figure 3:**
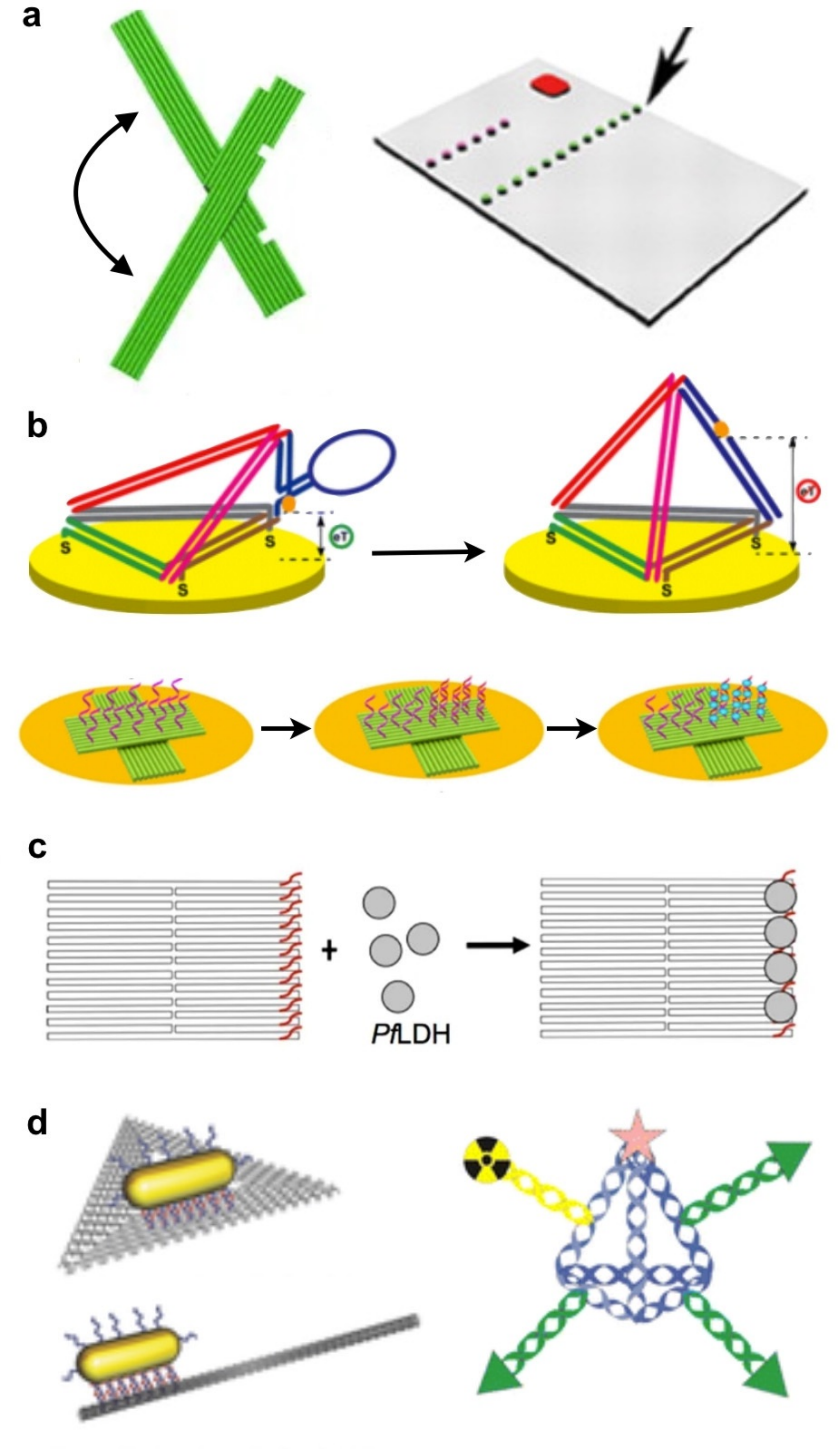
DN‐based diagnostics and imaging. a) DN for AFM‐based microRNA (miRNA) detection. Right panel: A rectangular DO template. Left panel: A dynamic plier‐like DO. b) Electrochemical nucleic‐acid detection using DN. Top panel: Switchable DT at a gold electrode. Bottom panel: DO‐based electrochemical miRNA platform. c) A DO for the aptamer‐based detection of a malaria protein biomarker. d) DN for imaging applications. Left panel: DO with gold nanorods for two‐photon luminescence. Right panel: A DT labeled with near‐infrared emitters and a radioactive Tc isotope. a) Right panel reproduced with permission from ref. 57. Copyright 2008 AAAS. Left panel reproduced with permission from ref. 59. Copyright 2011 Springer Nature. b) Top panel reproduced with permission from ref. 67. Copyright 2014 American Chemical Society. Bottom panel reproduced with permission from ref. 68 (https://pubs.acs.org/doi/10.1021/acsomega.9b01166). Copyright 2019 American Chemical Society. Further permissions related to the material excerpted should be directed to the American Chemical Society. c) Reproduced with permission from ref. 70. d) Left panel reproduced with permission from ref. 49. Copyright 2015 John Wiley and Sons. Right panel reproduced with permission from ref. 71. Copyright 2016 American Chemical Society.

Being fully composed of DNA, it is rather straightforward to decorate DN with a well‐defined arrangement of capture probes for the specific binding of preselected, medically relevant nucleic‐acid sequences such as cancer‐related microRNAs (miRNA)^56^ or disease‐ or pathogen‐specific genes. Target binding can be detected using various techniques, with the DN often being used for signal enhancement, transduction, or the implementation of logic operations. One of the earlier demonstrations by Ke et al. used barcoded DO substrates to arrange three different capture sequences complementary to regions of three different genes and detected the site‐specific binding of target RNAs using atomic force microscopy (AFM, Figure 3 a, right panel).^57^ Wang et al. further advanced this concept by implementing strand‐displacement‐based logic operations for the simultaneous detection of two different input miRNAs.^58^ Kuzuya et al. developed a dynamic plier‐like DO device that enabled the detection of several molecular species including miRNAs (Figure 3 a, left panel).^59^ Target binding resulted in a change in DO shape that could be detected either by AFM or by fluorescence readout using dye‐modified DO constructs.^60^

As of now, fluorescence‐based signal readout is the most widely used technique for the detection of target DNA/RNA binding to DN. This is mostly because the use of DN substrates for fluorophore presentation provides several advantages over free nucleic‐acid probes. For instance, DN enable the construction of multicolor‐fluorescence systems that can enter living cells for the detection of intracellular RNAs.^61^ Furthermore, DN provide several means for enhancing fluorescence intensities. Decoration of beacon‐like DO with multi‐fluorophore arrays resulted in enhanced FRET signals for target DNA detection down to 100 pm concentrations.^62^ Zhu et al. recently demonstrated the pH‐controlled intracellular release of hairpin probes from triple‐helix‐functionalized DT, which initiated a hybridization chain reaction upon interaction with target mRNA for the amplification of fluorescence signals.^63^ By modifying DO with gold or silver nanoparticles, plasmonic nanoantennae can be constructed for the plasmonic enhancement of the fluorescence signal obtained from single fluorophores by several orders of magnitude.^64^ Ochmann et al. combined such nanoantennae with fluorescence‐quenching hairpins to detect Zika‐virus DNA and RNA sequences.^65^ This approach not only enabled target detection at 1 nm concentrations in human serum but was also extended toward multiplexing by combining multiple antenna designs.

In addition to AFM and fluorimetry, various electrochemistry‐based sensing concepts for the detection of nucleic‐acid binding to electrode‐immobilized DN have also been evaluated (Figure 3 b).^66^, ^67^, ^68^ Here, immobilizing target‐binding probe sequences on electrode‐supported DN typically resulted in a higher detection sensitivity and sequence specificity compared to direct immobilization at the electrode surfaces.

Employing dynamic DN allows for the implementation of novel sensing concepts. Funck et al. recently demonstrated the detection of a hepatitis C virus RNA sequence using a cross‐shaped DO comprised of two gold nanorod‐carrying arms connected with a flexible pivot point.^69^ Target RNA binding triggered a strand‐displacement reaction that resulted in the switching of the DO device from a mostly achiral to a right‐handed chiral geometry, which could be detected by circular‐dichroism spectroscopy. The sensitivity of the device was determined as 100 pm and successful detection of 1 nm target RNA was accomplished in 10 % serum.

In a rather different approach, DO have been used as shape IDs not for DNA detection but for genotyping. To this end, Zhang et al. developed a set of differently shaped and modified DO that were used for the site‐specific labeling of genomic DNA extracted from human blood samples.^72^ Using AFM for shape ID visualization, the authors could detect and distinguish various single nucleotide polymorphisms (SNPs) with a lateral solution of about 10 nm. This approach was later extended to identify the genotype of hepatitis B viruses.^73^

The general detection concepts introduced above can also be adapted for the detection of proteins and other biomarkers. This usually requires the introduction of target‐specific aptamers into the DN.^74^ Detection of the bound analyte can then be achieved using AFM,^70^, ^75^ electrochemistry,^76^ fluorimetry,^60^ and CD spectroscopy,^77^, ^78^ and successful target detection was recently demonstrated even in whole blood.^76^ Nevertheless, most of these works can be considered proof‐of‐principle studies that employed well‐characterized aptamers with a high affinity toward some model targets such as thrombin,^75^, ^78^ ATP,^60^, ^76^ or adenosine.^77^ Successful aptamer‐based detection of an infection‐related target was demonstrated by Godonoga et al., who incorporated aptamers against the malaria‐protein biomarker *Pf*LDH in 2D DO substrates (Figure 3 c).^70^ Using AFM, target‐protein binding could be detected at a concentration as low as 500 nm and also in the presence of human plasma. Kwon et al. decorated star‐shaped DN with a regular pattern of aptamers that matched the expression pattern of their target proteins in the dengue‐virus envelope, resulting in strong oligovalent virus binding.^79^ By introduction of fluorophore‐quencher pairs, DN‐virus binding could be detected in human serum and plasma at a superior sensitivity compared to PCR‐based detection. Furthermore, arranging the aptamers on the DN also enhanced their inhibition efficacy.

As an alternative to aptamers, DN can also be directly modified to carry antibodies or antigens. For instance, Kuzuya et al. used fluorescein‐modified DO pliers for anti‐fluorescein IgG detection.^59^ Pei et al., on the contrary, developed a sandwich‐type assay in which an antibody against TNF‐α was attached to DT immobilized on a gold electrode.^80^ After capturing TNF‐α from solution, a second antibody carrying a HRP enzyme was bound to the captured TNF‐α to translate target binding into a detectable electrochemical signal. This sandwich‐type assay was subsequently further advanced toward the detection of antibodies^81^ and pathogens.^82^, ^83^ For instance, Wang et al. recently reported the highly sensitive detection of pneumococcal surface protein A from *Streptococcus pneumoniae* lysate.^83^ This assay not only had an extremely low limit of detection of 0.093 CFU mL^−1^ equivalent of *S. pneumoniae* lysate, but was also able to quantify *S. pneumoniae* in different swab samples from a human subject.

DN can also be employed as carriers of functional species for in‐vivo‐imaging applications.^84^ Many studies have employed fluorophore‐ and quantum‐dot‐labeled DN to visualize their biodistribution by fluorescence microscopy.^13^, ^39^, ^85^, ^86^ While most of these works focused on the efficacy of the DN as vehicles for the transport of therapeutic cargo, this approach may also find its way into purely diagnostic applications. For instance, Kim et al. used a fluorophore‐labeled DT for sentinel‐lymph‐node imaging.^87^ Here, the use of a DN resulted in enhanced lymph‐node translocation and a prolonged retention time at the node.

In addition to fluorescence imaging, DN have recently also been utilized in other in‐vivo‐imaging techniques. Jiang et al. employed a dual‐modified DT carrying a near‐infrared (NIR) emitter and a radioactive Tc isotope for combined NIR‐fluorescence imaging and single‐photon‐emission‐computed tomography of targeted tumors in mice (Figure 3 d, right panel).^71^ Similarly, the in‐vivo biodistribution of ^64^Cu‐labeled DO was evaluated using positron‐emission tomography.^55^ Finally, gold‐nanorod‐modified DO have been employed for two‐photon luminescence (Figure 3 d, left panel)^49^ and optoacoustic imaging.^50^

## 3 Challenges for Applying DNA Nanostructures in the Physiological Environment

The application of DN in physiological environments faces two major challenges: limited stability in biological media and the induction of an adverse immune response. On top of that, the structures usually suffer from poor cell uptake. Naturally, these challenges are more severe for in‐vivo applications than for ex‐vivo diagnostics. However, even though many ex‐vivo assays may use biological media that have been purified, inactivated, supplemented with stabilizing salts, or simply diluted, the ultimate goal is to directly analyze patient samples with as little pre‐processing as possible, in particular in the field of point‐of‐care diagnostics.^88^

### 3.1 Limited Stability

Early on, the stability of DN in biological media has attracted considerable attention and initial results appeared very promising. Keum and Bermudez have shown that DT are more stable in the presence of diluted serum than their linear counterparts.^89^ Similarly, Mei et al. demonstrated the stability of various 2D and 3D DO in cell lysates.^90^ Nevertheless, Castro et al. observed the complete degradation of 3D DO in the presence of nucleases in less than one hour.^91^ Finally, Hahn et al. have identified two major factors that limit DO stability in cell‐culture media, namely low Mg^2+^ concentrations and the presence of nucleases.^92^

The vast majority of protocols for DN assembly employ Mg^2+^ concentrations in the mm range in order to compensate the electrostatic repulsion between neighboring DNA helices.^93^ Relevant biological media such as blood or serum, however, have much lower Mg^2+^ concentrations.^92^ Even though physiologically more abundant monovalent cations such as Na^+^ or K^+^ are, in general, also able to stabilize the DN, they are less efficient and may again require concentrations that exceed those of physiological environments.^94^, ^95^ This is due to ion‐specific differences in the type of interaction, binding site, and binding affinity between DNA and the individual ion species.^94^, ^96^ Recently, Roodhuizen et al. performed atomistic molecular‐dynamics simulations of DO stabilization by various cations.^96^ They found that Mg^2+^ ions bind strongly to minor‐groove atoms and the backbone phosphates, whereas Na^+^ binding is much weaker and barely involves the phosphates. This strong binding of the Mg^2+^ ions is responsible for the somewhat surprising observation that DO assembled in a Mg^2+^‐containing buffer can be gently transferred into Mg^2+^‐free buffers and even pure water without a loss of integrity.^94^, ^97^ Under such conditions, however, other buffer components such as EDTA or phosphate ions also become important as they may interfere with the DNA‐bound Mg^2+^ ions and thus promote DO denaturation.^94^ Furthermore, DO shape and superstructure also appear to play a significant role in DO stability and denaturation under low‐ionic‐strength conditions.^92^, ^94^

While these ionic‐strength‐related destabilizing effects are typically more pronounced in large DO that feature a dense arrangement of many negatively charged double helices than in smaller DN,^98^ DT^99^ and single‐stranded‐tile (SST)‐based DNA nanotubes^100^ may also turn out sensitive toward low mono‐ and divalent salt concentrations. In particular, Kocabey et al. observed that SST‐based DNA nanotubes may undergo complete denaturation in phosphate‐buffered saline with a physiological Na^+^ concentration of 135 mm when supplemented with less than 2 mm Mg^2+^.^101^ Even more intriguing, they found that low‐salt denaturation is more pronounced when siRNA sequences are hybridized to single‐stranded overhangs on the nanotube surface.

DNA‐degrading nucleases are found in virtually all types of tissues and bodily fluids,^102^, ^103^ and thus represent a serious threat to the integrity of therapeutic or diagnostic DN. Consequently, a number of studies have evaluated DN stability in the presence of selected nucleases or serum‐containing media. Unfortunately, the obtained results remain somewhat ambiguous so far. While Keum and Bermudez observed that DT are more stable in 10 % fetal bovine serum (FBS) and in the presence of DNase I than simple double‐stranded DNA,^89^ Goltry et al. have shown that the situation is more complex.^104^ In particular, they observed not only that the stability of a simple DNA nanomachine in 70 % human serum depends on its local topology, but also that it was in general less stable than corresponding duplex DNA. Furthermore, the introduction of fluorophore and quencher labels resulted in a reduced stability of both the nanomachine and the duplex DNA. This observation is in line with the results by Kocabey et al., who found that siRNA‐decorated DNA nanotubes were degraded in 10 % FBS within 1 h, whereas unmodified nanotubes survived for at least 8 h under identical conditions.^101^ As was recently shown by Lacroix et al., this rapid enzymatic degradation of fluorescently labeled DN in cell‐culture medium may cause severe artifacts in in‐vitro cellular‐uptake studies.^105^ For larger DO, exposure to 10 % FBS resulted in notable degradation already after two hours.^92^ This fast degradation is caused by the very high nuclease activity of FBS. In human serum, about sixfold longer lifetimes have been observed.^104^ Using FBS, the lower nuclease activity of human serum can be mimicked by heat inactivation,^104^ which resulted in completely intact DO after 24 h incubation.^92^

Castro et al. investigated the susceptibility of 3D DO‐helix bundles toward degradation by various nucleases and observed significant digestion only for T7 endonuclease I and, most importantly, DNase I,^91^ which is the most abundant nuclease in blood and serum.^102^ Nevertheless, DNase‐I‐induced degradation was much slower for the DO than for duplex plasmid DNA.^91^ Absolute time scales of DNase‐I‐induced DO degradation, however, strongly depend on the experimental conditions. For instance, Castro et al. observed the complete digestion of a DO 24‐helix bundle (2 ng in 20 μl) by one unit DNase I within 1 h at 37 °C.^91^ Auvinen et al., on the contrary, observed no degradation at all for a 60‐helix bundle (310 ng in 20 μl) exposed to one unit DNase I within 1 h at room temperature.^106^ These differences may result not only from the different temperatures and DNA concentrations employed in these experiments, but also from the different DO shapes. Indeed, as was recently shown by our labs, DO shape and superstructure play an important role in modulating global as well as local DNase‐I activity (see Section 4.1).^107^

### 3.2 Adverse Immune Response

Albeit DNA molecules are inherently biocompatible polymers and act as key players in many biological and cellular processes, they may nonetheless elicit severe inflammatory responses.^108^ In the case of DN, Perrault and Shih^85^ as well as Auvinen et al.^106^ observed remarkable immune activation in mice splenocytes incubated with plain 3D DO just by monitoring their cytokine production, namely interleukin 6 (IL‐6) and/or interleukin 12 (IL‐12) levels. Nevertheless, when these primary spleen cells were treated with lipid‐ or protein‐coated DO (see also Section 4.2), both groups demonstrated a proper camouflage of DNA, in other words, a negligible immune response. Schüller et al. (see also Section 2.1) also probed the immune response of mice splenocytes, but in this case, they used tubular DO with and without immunostimulatory CpG oligonucleotides and followed the secretion of three different immune markers, IL‐6, IL‐12, and transmembrane C‐type lectin (CD69).^44^ Cell incubation with CpG‐modified tubes resulted in elevated levels of all markers, while unmodified DO induced only IL‐6 and IL‐12 responses. In contrast, a study by Xia et al. employing dendritic RAW264.7 cells (mouse‐macrophage‐precursor immune cells) and small DT did not show any IL‐6 or IL‐12 activation.^109^ Nevertheless, the direct comparison of these results is rather challenging, as the amount and size of DN vary from study to study.^110^

## 4 Strategies and Solutions

### 4.1 Design Factors

**Figure 4:**
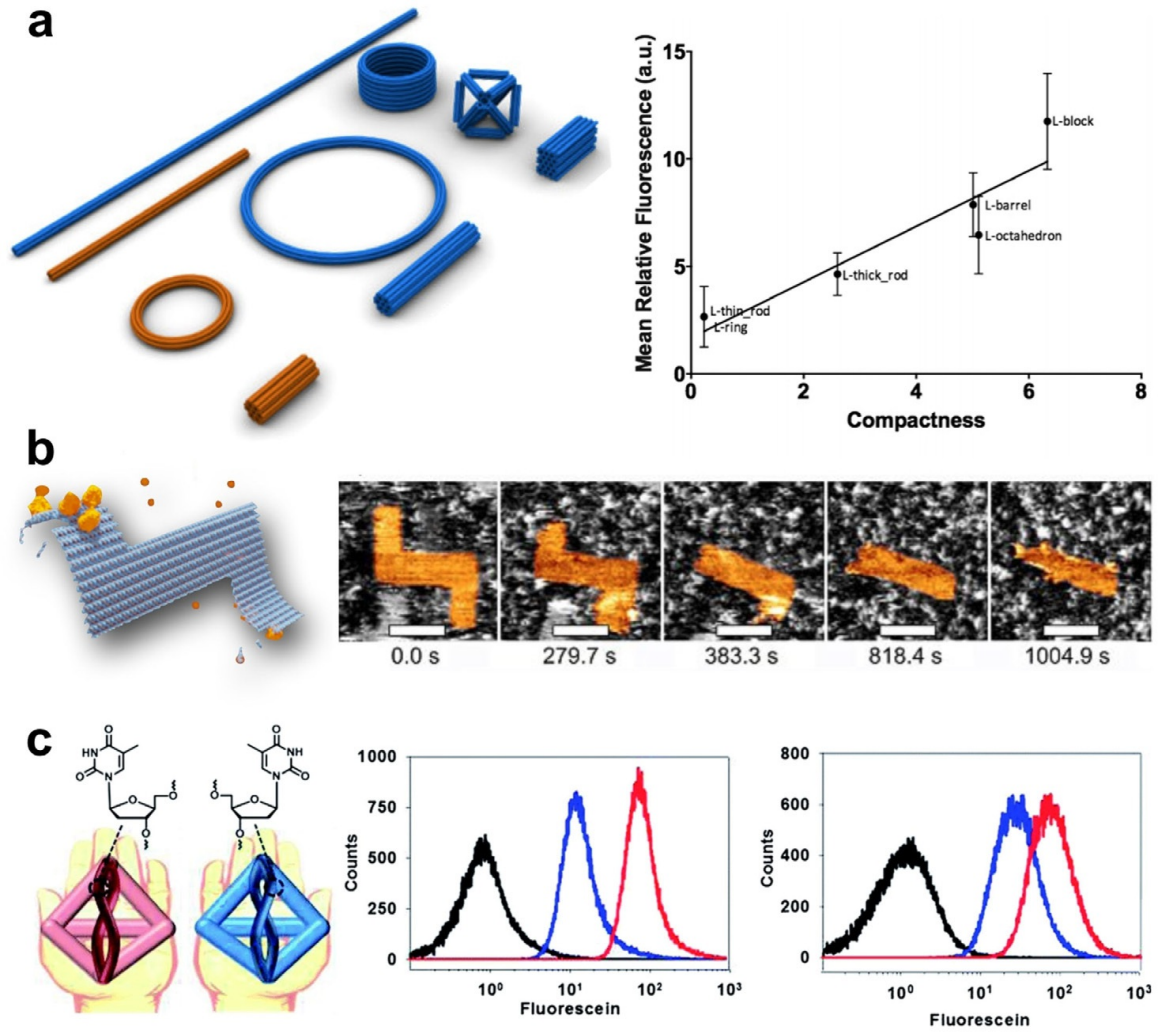
Design factors that affect DN delivery and stability. a) Left panel: DO with varied shapes and masses for studying their cellular uptake. Right panel: The results indicate enhanced delivery with increasing compactness of the DN. b) DO under DNase‐I attack analyzed in real time using high‐speed AFM. c) DT with bioorthogonal base‐pairing systems, l‐DT (red) design enhances HeLa (left) and NIH‐3T3 (right) intracellular delivery and outperforms d‐DT (blue), as shown by flow cytometry (untreated cells shown in black). a) Reproduced with permission from ref. 111. Copyright 2018 American Chemical Society. b) Reproduced with permission from ref. 107. Copyright 2019 John Wiley and Sons. c) Reproduced with permission from ref. 113.

DN can be designed in a variety of shapes, and for a long time, it was speculated that their shape and size should have a significant influence on the efficacy of their cellular transport, similar to nanocarriers made of other materials. Bastings et al. thus systematically studied the living‐cell uptake of DO having a size range of 2 to 5 MDa and multiple structures such as different‐diameter bundles, circles, barrels, and other nanoshapes (Figure 4 a, left panel).^111^ They observed that large and compact structures are preferentially internalized (Figure 4 a, right panel), indicating that both the aspect ratio and mass play an important role in the process. Nevertheless, they reported that the transfection rates are actually even more dependent on the used cell type than the DO themselves.

In any case, these structures will eventually face the adaptive immune system (see above) and endonucleases, which may digest and eventually destroy them. Keum and Bermudez have reported that simple DT with different shapes and sizes may be resistant to specifically and non‐specifically digesting nucleases.^89^ Kim et al. used a DNA cage library of 16 different shapes for the in‐vivo screening of tumor targeting in mice and found that the tumor specificity was closely related not only to the cellular uptake of the cages but also to their nuclease resistance.^112^ By selecting the most potent cages, the group demonstrated tumor‐specific damage by delivering macromolecular apoptotic proteins solely into the tumor tissue.

Ramakrishnan et al. studied how DNase I would enzymatically cleave multiple distinctly designed DO by monitoring the structures in real time on mica substrates using high‐speed AFM.^107^ They observed that these 2D shapes exhibit structure‐specific degradation profiles, thus confirming that digestion is structure‐dependent (Figure 4 b). In particular, DO degradation by DNase I seems to be most efficient at mechanically flexible sites that can accommodate the structural duplex distortions associated with DNase‐I binding. Similar observations were also made by Stopar et al. who investigated the enzymatic cleavage of the DO scaffold by several restriction endonucleases at their respective recognition sites.^114^ They found that these sites can be either active or strongly resistant toward enzymatic cleavage, depending on the local mechanical and topological properties in the vicinity of the individual site.^114^, ^115^

The bulk of these data suggests that DN can be rationally designed for increased serum stability and cell uptake, for instance, by making them mechanically very rigid to suppress nuclease binding. However, the desired performance of a given DN depends not only on its stability and uptake but also on other aspects such as drug loading efficiency and release kinetics, which are affected by the same design factors. Individual design‐related properties will thus have to be weighed against each other, favoring, for instance, efficient drug intercalation over serum stability.

As an interesting addition to the different designs discussed above, Kim et al. employed a bio‐orthogonal base‐pairing system, that is, l‐DNA instead of natural d‐DNA, for their DT vehicle to avoid undesirable interaction between the carrier and the attached proliferative aptamer cargo (Figure 4 c).^113^ This modification also led to strongly enhanced serum stability and increased intracellular delivery rates. Similarly, Liu et al. introduced unnatural base pairs into DNA junctions and nanotubes, resulting in increased melting temperatures and exonuclease resistance.^116^ It remains to be seen, however, how such oligonucleotide modifications affect the loading with intercalating or groove‐binding drugs.

### 4.2 External Modifications

**Figure 5:**
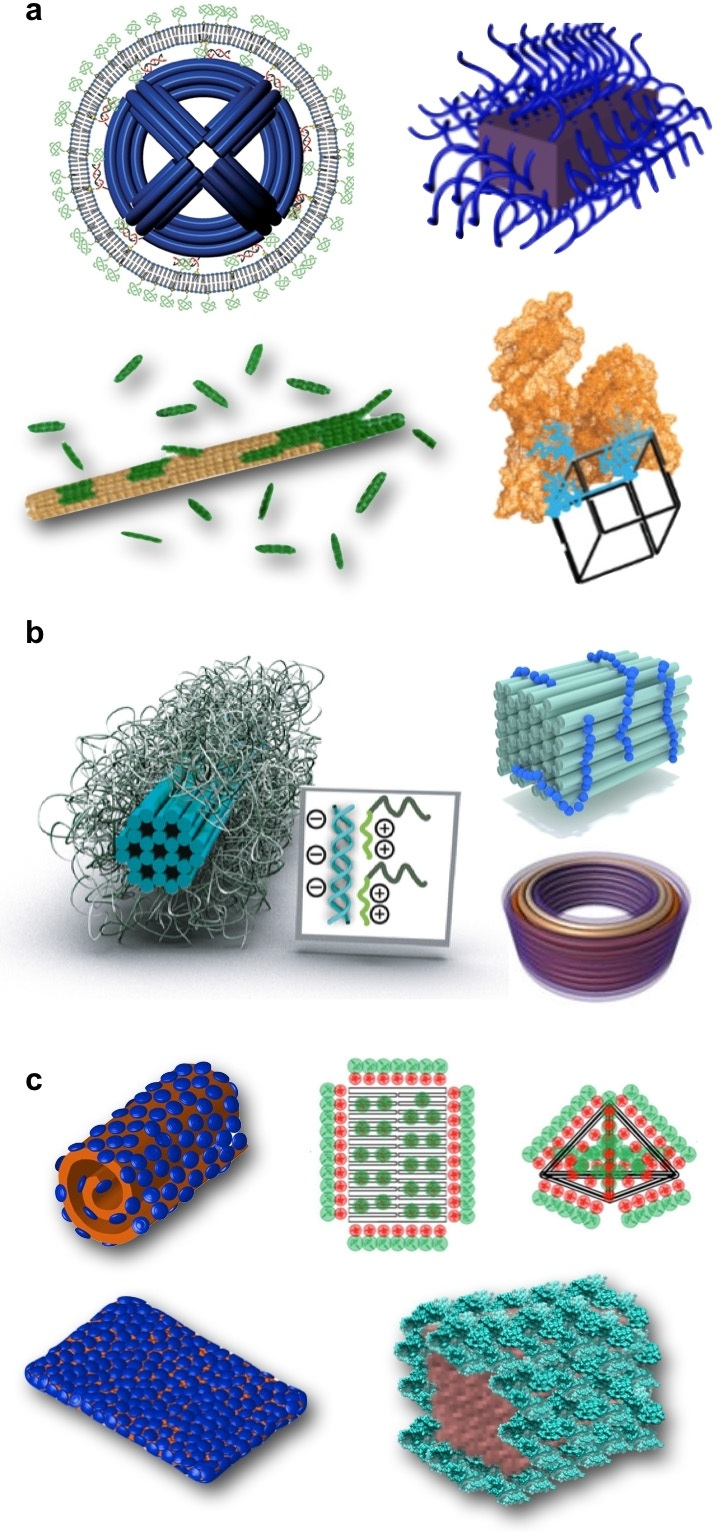
External modifications of DN. a) Coating and healing strategies for various DNA shapes using direct DNA linking. Top left panel: A sphere‐like DO with virus‐inspired lipid coating. Top right panel: A dendritic oligo‐coated DNA brick. Bottom left panel: A DNA nanotube that self‐heals in serum. Bottom right panel: A HSA‐equipped DNA cube. b) Electrostatic polymer coating of DO. Left panel: Reversible cationic polymer coating of a DNA bundle. Top right panel: A cationic polymer‐coated DNA brick. Bottom right panel: An oligolysine‐coated barrel‐like DO. c) Peptide and protein‐coated DN. Left panels: A rectangular DO complexed with virus capsid proteins yielding different morphologies. Top right panel: Rectangular and tetrahedral DN with diblock polypeptides. Bottom right panel: A bovine serum albumin (BSA)‐coated brick‐like DO. a) Top left panel reproduced with permission from ref. 85 (https://pubs.acs.org/doi/10.1021/nn5011914). Copyright 2014 American Chemical Society. Further permissions related to the material excerpted should be directed to the American Chemical Society. Top right panel reproduced with permission from ref. 117. Bottom left panel reproduced with permission from ref. 119. Copyright 2019 American Chemical Society. Bottom right panel reproduced with permission from ref. 120. Copyright 2017 American Chemical Society. b) Left panel reproduced with permission from ref. 121. Copyright 2017 John Wiley and Sons. Top right panel reproduced with permission from ref. 28. Bottom right panel reproduced with permission from ref. 86. c) Left panel reproduced with permission from ref. 122. Copyright 2014 American Chemical Society. Top right panel reproduced with permission from ref. 123. Copyright 2017 American Chemical Society. Bottom right panel reproduced with permission from ref. 106.

The most intuitive way to tackle possible immunogenicity, poor transfection rates, and low stability is to employ coating or self‐healing strategies. Perrault and Shih demonstrated that the DNA strand‐mediated lipid‐bilayer coating of a spherical DO not only attenuated the immune response (see Section 3.2), but also improved the stability against nucleases and increased the in‐vivo pharmacokinetic bioavailability (Figure 5 a, top left panel).^85^ The stability and resistance to nucleases can also be increased by decorating the exterior of DN with dendritic oligonucleotides as shown by Kim and Yin (Figure 5 a, top right panel).^117^ Lacroix et al. used a human‐serum‐albumin (HSA) coating based on the attachment of dendritic alkyl‐conjugated DNA strands for achieving serum stability and protection against nucleases (Figure 5 a, bottom right panel).^120^ It is noteworthy that the employed HSA coating did not hinder the activity of structure‐bound gene‐silencing oligonucleotides inside cells. In addition to the study by Lacroix et al., it has also been shown that discrete protein modifications can enhance the cellular delivery of DN. Schaffert et al. incorporated multiple DNA‐modified transferrin proteins into a planar DO and managed to increase the transport rates into cancer cells up to 22‐fold compared to naked DO.^118^ Yet another direct DNA‐linking‐based approach to increase the bioavailability of DN is self‐healing (Figure 5 a, bottom left panel). Li and Schulman demonstrated that the degradation process of long poly(ethylene glycol) (PEG)‐coated DNA nanotubes in 10 % FBS could be reversed by an excess amount of small PEG‐conjugated DNA tiles to seal the broken parts and repair defects.^119^ With this strategy, the serum lifetime of DNA nanotubes could be extended to several days.

All the coating approaches mentioned above rely on modified DNA strands that can be anchored via hybridization to the designed positions in the larger structure, where they may equally serve as binding sites for further functionalization. While the addressability is arguably an advantage, these methods may require expensive modifications of dozens of individual strands. Therefore, instead of DNA conjugation, simple electrostatic coating and shielding of the negatively charged DN may be more feasible in many occasions. There is a plethora of techniques that take advantage of cationic polymers, such as poly(2‐dimethylaminoethyl methacrylate) (PDMAEMA)–PEG copolymers (Figure 5 b, top right panel),^28^ chitosan and linear polyethyleneimine (PEI),^124^ oligolysine–PEG (Figure 5 b, bottom right panel),^86^ and polylysine–PEG copolymers (Figure 5 b, left panel).^121^ Recently, it was demonstrated that the nuclease resistance of DO coated with PEGylated oligolysine can be further enhanced by a chemical crosslinking of the polymer coating.^125^

Besides synthetic polymers, it is equally possible to apply protein‐based coatings using electrostatic interactions. Mikkilä et al. disassembled chlorotic cowpea mottle virus (CCMV) to obtain single capsid proteins (CPs) with positively charged residues at the N‐terminus.^122^ These CPs were then complexed with rectangular DO, resulting in wrapped‐up morphologies (Figure 5 c, left panels) or fully CP‐encapsulated DO. The HEK 293 cell‐transfection efficiency of CP‐coated DO could be increased up to 13‐fold compared to bare DO. Kopatz et al. used a similar strategy as above, but their components were CPs of simian virus 40 (SV40) from the polyomavirus family and a nearly spherical 3D DO with a well‐defined diameter.^126^ In this case, CPs were fully encapsulating the DO, and the complex resembled the native SV40 in shape, symmetry, and size. In addition to viral capsids, serum albumin has also been used for the protection of DO. Auvinen et al. demonstrated the bovine serum albumin (BSA) coating of a brick‐like DO by covalently coupling positively charged and branched dendron molecules to BSA (Figure 5 c, bottom right panel).^106^ It was not only shown that the BSA corona attenuated the immune response as explained earlier, but also that the cell‐transfection rates and stability against DNase I were clearly improved. Recently, cationic HSA (cHSA), an albumin derivative, was utilized in a similar way to complex rectangular DO.^127^ Yet another way of making use of protein‐based shielding is to attach protein polymers^128^ or diblock polypeptides^123^ to DN to protect them from enzymatic degradation (Figure 5 c, top right panel).

Several issues need to be considered when employing such coatings in biomedical DN. While it has been demonstrated that oligolysine–PEG coating of DO did not compromise the functionality of single‐stranded DNA handles protruding from the DO surface,^86^ this may not be the case for all coatings and functional surface modifications reported in literature. In particular, the binding affinity of surface‐bound aptamers may be drastically reduced by the application of such coatings. Furthermore, many of the coatings discussed above are not compatible with the conformational switching of dynamic DN. Finally, coating of DN will restrict access to any encapsulated cargo^28^ and thus (positively or negatively) affect drug‐loading and release properties.

### 4.3 Enzymatic and Chemical Modifications

A general problem faced by virtually all DN results from the fact that they consist of short oligonucleotides that are hybridized with each other via domains comprising only a small number of base pairs. Therefore, the melting temperatures of these oligonucleotides are often rather low. Furthermore, even a moderate nuclease attack resulting in only a few cleaved oligonucleotides may already lead to the spontaneous dehybridization of the even shorter fragments and thus the complete collapse of the DN. Consequently, various studies have attempted to increase DN stability by introducing covalent bonds between neighboring oligonucleotides.

**Figure 6:**
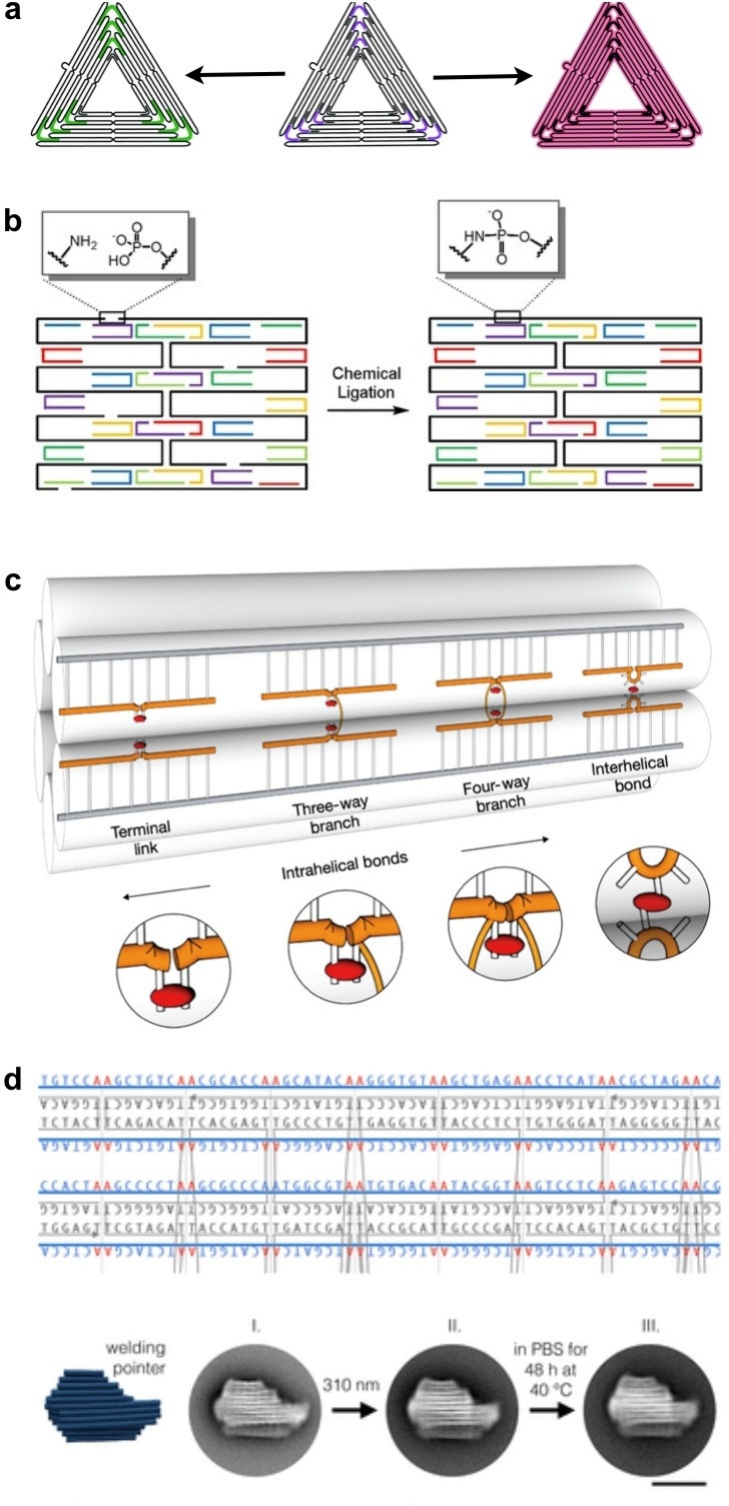
Enzymatic and chemical modifications of DN. a) Redesigned (left) and ligated (right) DO triangles for enhanced stability. b) Chemical ligation of a rectangular DO tile. c) DO staples are crosslinked by forming covalent UV‐induced cyclobutane pyrimidine dimers between thymine bases. d) Top panel: Combination of design and chemistry: a custom scaffold allows tailored UV‐crosslinking throughout a DO. Bottom panel: After the UV‐treatment the DO pointer structure retains its shape when incubated for 48 h in low ionic strength phosphate‐buffered saline (PBS) at 40 °C. a) Reproduced with permission from ref. 130. b) Reproduced with permission from ref. 133. Copyright 2014 John Wiley and Sons. c) Reproduced with permission from ref. 135. d) Reproduced with permission from ref. 136. Copyright 2019 American Chemical Society.

A rather obvious strategy for the covalent linking of (selected) oligonucleotides is their phosphorylation and post‐assembly enzymatic ligation. O'Neill et al., for instance, demonstrated that ligation of tile‐based DNA nanotubes not only increases their melting temperature but also renders them stable in pure water.^129^ Even though the dense duplex arrangement in DO most likely makes a significant fraction of nicks non‐accessible for the ligase,^107^, ^114^ the enhanced stability of ligated DO under denaturing conditions was recently demonstrated (Figure 6 a).^130^ Hamblin et al. further demonstrated that decreasing the number of backbone nicks renders DNA nanotubes resistant against nuclease degradation in 10 % FBS.^131^ In their case, this was achieved not by enzymatic ligation but through the use of continuous backbone strands produced by rolling‐circle amplification.

Similar covalent crosslinks can also be achieved via chemical modifications of the employed oligonucleotides. Casinelli et al. assembled SST‐based six‐helix tubes from (3′‐alkyne,5′‐azide)‐modified oligonucleotides.^132^ Each of these oligonucleotides then was cyclized by covalently connecting its ends in a post‐assembly click reaction, which resulted in the topological interlocking of the SSTs. This interlocking resulted in a significantly enhanced stability of the DNA nanotubes under low‐salt conditions and enhanced resistance against exonuclease digestion. Kalinowski et al. followed a different approach and chemically ligated the oligonucleotides in a DO via phosphoramidate linkages between 3′‐amino‐modified staples and their 5′‐phosphorylated neighbors (Figure 6 b).^133^ Recently, Raniolo et al. evaluated the stability of various crosslinked and non‐crosslinked DN both in 10 % FBS and inside cells.^134^ They found that enzymatic ligation of DNA cages as well as cyclization of SST‐based DNA nanotubes resulted in a significantly enhanced stability compared to both their non‐crosslinked counterparts and non‐ligated DO.

In addition to these purely chemical crosslinking strategies, various routes for the photo‐induced crosslinking of DO have been reported. Rajendran et al. incubated pre‐assembled DO with the drug 8‐methoxypsoralen (8‐MOP).^137^ Subsequent exposure to UV light resulted in the formation of covalent adducts between 8‐MOP and pyrimidine bases. The UV‐crosslinked DO exhibited drastically enhanced melting temperatures. Gerling et al. employed a more direct photo‐crosslinking.^135^ They modified the staple strands with single‐stranded thymidines at predefined positions and employed UV irradiation to crosslink thymidines in close proximity via the formation of cyclobutane pyrimidine dimers (CPDs). In this way, they introduced various interhelical as well as some intrahelical crosslinks (Figure 6 c). The UV‐crosslinked DO displayed superior stability under high‐temperature and low‐salt conditions as well as in 10 % FBS and in the presence of various nucleases. Engelhardt et al. further advanced this approach by utilizing a specifically designed scaffold with AA motifs at all possible staple crossover positions, which therefore leads to adjacent thymidines in staple strands that can be readily UV‐crosslinked without requiring any additional staple modifications (Figure 6 d).^136^ Finally, photo‐crosslinking can also be reversible, as recently demonstrated by Gerling and Dietz, who employed 3‐cyanovinylcarbazole‐modified staple strands in DO.^138^ Upon irradiation with UV light at 365 nm, this modification can form a covalent bond with a thymine base in its close vicinity. This bond can be reversibly cleaved upon irradiation at a 310 nm wavelength, which enables the transient stabilization of switchable DO devices.

Covalent crosslinking strategies as described above have proven very successful in stabilizing DN under physiological and denaturing conditions. However, there are certain issues associated with the crosslinking concept in general that may somewhat limit the application of these strategies in biomedical settings. For instance, in many cases, covalent crosslinking may lock dynamic DNA devices in a fixed conformation and thus inhibit any switching action in response to a detected stimulus. Furthermore, crosslinking may alter the mechanical properties of individual duplexes and thereby affect drug‐loading and release properties. In this regard, the impact of covalent crosslinking on the performance of a given DN is rather difficult to predict and needs to be assessed individually in each application.

As an alternative to covalent crosslinking, non‐crosslinking‐based strategies may also be able to enhance DN stability. Conway et al., for instance, demonstrated an improved serum stability of prism‐like DNA cages assembled from oligonucleotides carrying terminal hexaethylene glycol and hexanediol modifications.^139^ In particular, the introduction of hexaethylene glycol modifications increased the lifetime of the DNA cages in 10 % FBS from about 2 h to about 15 h. This strategy has the great advantage that interference with structural properties and cargo accessibility is kept at a minimum. Whether these modifications are also able to stabilize larger DO, however, remains to be seen.

## 5 Summary and Outlook

Folding DNA into customized shapes with desired functions is no more just a black‐box system, since we continuously gain more knowledge of its inner workings. This progress has enabled better designs, faster production, higher fabrication yields, and in some cases, more stable structures. As a result, DN have found their way into numerous application fields. In particular, the field of biomedical DNA nanotechnology has seen immense advancements in the last decade, with DN being employed in numerous applications, ranging from pathogen detection to genotyping to drug delivery and targeted therapy. Several successful tumor treatments in animal models have been demonstrated using DN delivery vehicles, and DN have even shown the potential to be used as active therapeutics themselves. Nevertheless, the limited stability under physiological conditions and possible immunogenicity of DN have raised concerns regarding impaired functionality and insufficient biodistribution and circulation time on top of undesired side effects.

Consequently, more and more research efforts focus on elucidating and controlling the molecular mechanisms that govern DN stability and degradation under physiological conditions. Several routes toward controlling DN stability and immunogenicity have already been explored, including rational (re)design strategies, enzymatic ligation, chemical and photo‐crosslinking, protein and lipid encapsulation, and polyelectrolyte coatings. While many of those approaches have indeed resulted in significant improvements in DN stability, biodistribution, and cellular uptake, they may also carry the risk of interfering with the therapeutic performance of the DN, primarily with the loading and release of therapeutic cargo and the binding of diagnostic biomarkers. Furthermore, many of the stabilization strategies investigated so far are incompatible with a dynamic switching of the DN, as used in several stimuli‐responsive DNA nanorobots. Tailoring DN stability, immunogenicity, dynamic switching, cargo loading and release, and analyte binding both simultaneously and independently thus represents the most eminent challenge that biomedical DNA nanotechnology currently faces.

Another challenge that we did not discuss so far, even though it will become a highly pertinent question at some point in the future, regards the clinical translation of therapeutic DN. Important subjects in this respect include long‐term storage and shelf‐life, scalability, CMC‐ and GMP‐compliant production, and cost. A number of studies have already addressed the issues of long‐term storage of DN and their pre‐assembled components by lyophilization and cryo‐storage, and further identified appropriate conditions to keep them intact for up to several years.^99^, ^140^ With regard to scalability and cost, previous in‐vivo studies using intravenous administration of DOX‐loaded DN typically applied doses in the range of about 100–600 μg DN per kg animal.^10^, ^13^, ^14^ For a human with a body weight of 75 kg, this would translate to a about 10–50 mg DN per dose. Employing biotechnological mass production, DO can be produced at an estimated €0.18 per mg,^141^ resulting in total DNA costs of less than €10 per dose. This number, however, does not include additional costs arising from CMC‐ and GMP‐compliant production, which may be rather challenging for biologics concerning issues of sterilization, purity, and batch‐to‐batch consistency,^142^ and thus increase the production costs significantly. However, there may also be additional regulatory matters. While several nucleic‐acid‐based drugs have been approved by the FDA and are already in clinical use,^143^ these comprise only synthetic oligonucleotides without any genomic material. Therefore, we expect that fully synthetic DN such as DT and SST‐based DN will have to overcome fewer hurdles on their way toward FDA approval than DO that are based on a genomic scaffold. Such DN also present the advantage that they can be assembled from other DNA‐like materials that are not potentially genetically active.^113^ While GMP‐compliant oligonucleotides are significantly more expensive than standard ones, large‐scale synthesis in the multi‐kg range will reduce the price to a few euros per mg, which is comparable to monoclonal antibodies. This would raise above estimate to a few €10 per dose, which is fairly moderate for a biopharmaceutical. For instance, under the above considerations, one dose of Trastuzumab in the AC‐TH regimen for the treatment of HER2+ breast cancer costs around $900,^144^ while the antisense oligonucleotide drug Nusinersen for the treatment of spinal muscular atrophy is prized at $125,000 per injection.^143^ Therefore, for therapeutic DN to enter clinical trials, significant investments will be required due to the comparatively large costs of small‐scale GMP‐compliant oligonucleotide synthesis. However, once a DN‐based drug formulation receives regulatory approval and enters the market, we expect production costs as well as the final product prices to drop to reasonable levels, thus rendering DN promising therapeutics for the treatment of numerous diseases.

## Conflict of interest

The authors declare no conflict of interest.

## Biographical Information

*Adrian Keller studied engineering physics at the University of Applied Sciences Coburg, Germany, and received his Ph.D. degree in physics from TU Dresden, Germany, in 2009. After two postdoc stays at Aarhus University, Denmark, and the Helmholtz‐Zentrum Dresden‐Rossendorf, Germany, he joined Paderborn University in 2014 where he established the Nanobiomaterials group at the Chair of Technical and Macromolecular Chemistry. He received his habilitation in chemical engineering of biomolecular systems in 2017 from Paderborn University. His research focuses on DNA nanotechnology, amyloid aggregation, and biointerfaces*.

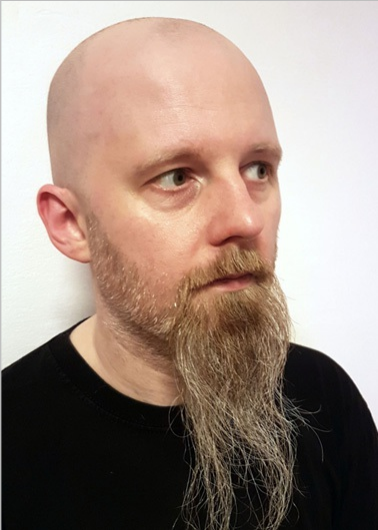

## Biographical Information

*Veikko Linko studied physics and chemistry at the University of Jyväskylä (JYU), Finland, and received his Ph.D. degree in physics in 2011. He carried out postdoctoral research in Prof. Hendrik Dietz's lab at TU Munich, Germany (2011–2013), and after that he has co‐led a DNA nanotechnology research line in Prof. Mauri Kostiainen's Biohybrid Materials group at Aalto University, Finland. He holds two docentships; one in physics (molecular nanotechnology, JYU, 2015) and one in chemical engineering (bionanotechnology, Aalto, 2018). His research focuses on structural DNA nanotechnology and its diverse applications in nanofabrication, biophysics and ‐chemistry*.

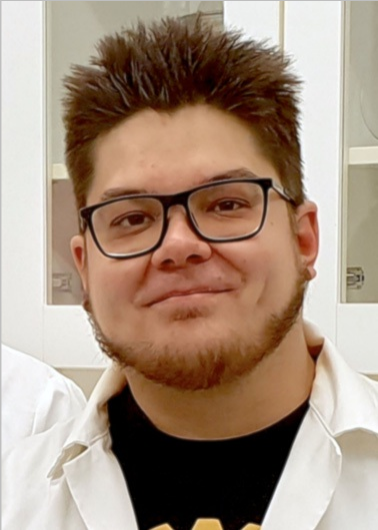

## References

[1a] M. Bathe , P. W. K. Rothemund , MRS Bull. 2017, 42, 882;

[1b] M. R. Jones , N. C. Seeman , C. A. Mirkin , Science 2015, 347, 1260901. 25700524 10.1126/science.1260901

[2a] H. Ijäs , S. Nummelin , B. Shen , M. A. Kostiainen , V. Linko , Int. J. Mol. Sci. 2018, 19, 2114; 10.3390/ijms19072114 PMC6073283 30037005

[2b] P. Wang , T. A. Meyer , V. Pan , P. K. Dutta , Y. Ke , Chem 2017, 2, 359.

[3a] S. Nummelin , J. Kommeri , M. A. Kostiainen , V. Linko , Adv. Mater. 2018, 30, 1703721; 10.1002/adma.201703721 29363798

[3b] M. DeLuca , Z. Shi , C. E. Castro , G. Arya , Nanoscale Horiz. 2020, 5, 182.

[4a] V. Linko , H. Dietz , Curr. Opin. Biotechnol. 2013, 24, 555; 23566376 10.1016/j.copbio.2013.02.001

[4b] F. Hong , F. Zhang , Y. Liu , H. Yan , Chem. Rev. 2017, 117, 12584. 28605177 10.1021/acs.chemrev.6b00825

[5a] Y. Wang , A. Santos , A. Evdokiou , D. Losic , J. Mater. Chem. B 2015, 3, 7153; 32262822 10.1039/c5tb00956a

[5b] P. Khanna , C. Ong , B. H. Bay , G. H. Baeg , Nanomaterials 2015, 5, 1163; 28347058 10.3390/nano5031163 PMC5304638

[5c] H. C. Fischer , W. C. W. Chan , Curr. Opin. Biotechnol. 2007, 18, 565. 18160274 10.1016/j.copbio.2007.11.008

[6a] E. S. Andersen , M. Dong , M. M. Nielsen , K. Jahn , R. Subramani , W. Mamdouh , M. M. Golas , B. Sander , H. Stark , C. L. P. Oliveira , et al., Nature 2009, 459, 73; 19424153 10.1038/nature07971

[6b] H. Dietz , S. M. Douglas , W. M. Shih , Science 2009, 325, 725; 19661424 10.1126/science.1174251 PMC2737683

[6c] S. M. Douglas , H. Dietz , T. Liedl , B. Högberg , F. Graf , W. M. Shih , Nature 2009, 459, 414; 19458720 10.1038/nature08016 PMC2688462

[6d] P. W. K. Rothemund , Nature 2006, 440, 297. 16541064 10.1038/nature04586

[7a] F. V. Reddavide , M. Cui , W. Lin , N. Fu , S. Heiden , H. Andrade , M. Thompson , Y. Zhang , Chem. Commun. 2019, 55, 3753; 10.1039/c9cc01429b 30860533

[7b] C. Kielar , F. V. Reddavide , S. Tubbenhauer , M. Cui , X. Xu , G. Grundmeier , Y. Zhang , A. Keller , Angew. Chem. Int. Ed. 2018, 57, 14873; 10.1002/anie.201806778 30216608

[7c] M. H. Hansen , P. Blakskjaer , L. K. Petersen , T. H. Hansen , J. W. Højfeldt , K. V. Gothelf , N. J. V. Hansen , J. Am. Chem. Soc. 2009, 131, 1322. 19123795 10.1021/ja808558a

[8a] D. Balakrishnan , G. D. Wilkens , J. G. Heddle , Nanomedicine 2019, 14, 911; 30901300 10.2217/nnm-2018-0440

[8b] Q. Hu , H. Li , L. Wang , H. Gu , C. Fan , Chem. Rev. 2019, 119, 6459; 29465222 10.1021/acs.chemrev.7b00663

[8c] Q. Jiang , S. Liu , J. Liu , Z.-G. Wang , B. Ding , Adv. Mater. 2019, 31, 1804785; 10.1002/adma.201804785 30285296

[8d] S. Liu , Q. Jiang , Y. Wang , B. Ding , Adv. Healthcare Mater. 2019, 8, 1801658; 10.1002/adhm.201801658 30938489

[8e] S. Mishra , Y. Feng , M. Endo , H. Sugiyama , ChemBioChem 2020, 21, 33; 31692184 10.1002/cbic.201900481

[8f] M. Nishikawa , M. Tan , W. Liao , K. Kusamori , Adv. Drug Delivery Rev. 2019, 147, 29; 10.1016/j.addr.2019.09.004 31614168

[8g] A. Udomprasert , T. Kangsamaksin , Cancer Sci. 2017, 108, 1535. 28574639 10.1111/cas.13290 PMC5543475

[9] A. Comberlato , K. Paloja , M. M. C. Bastings , J. Mater. Chem. B 2019, 7, 6321. 31460563 10.1039/c9tb01222b

[10] J. H. Kang , K.-R. Kim , H. Lee , D.-R. Ahn , Y. T. Ko , Colloids Surf. B 2017, 157, 424. 10.1016/j.colsurfb.2017.06.014 28645043

[11] C. Wiraja , Y. Zhu , D. C. S. Lio , D. C. Yeo , M. Xie , W. Fang , Q. Li , M. Zheng , M. van Steensel , L. Wang , et al., Nat. Commun. 2019, 10, 1147. 30850596 10.1038/s41467-019-09029-9 PMC6408537

[12] Y.-X. Zhao , A. Shaw , X. Zeng , E. Benson , A. M. Nyström , B. Högberg , ACS Nano 2012, 6, 8684. 22950811 10.1021/nn3022662

[13] Q. Zhang , Q. Jiang , N. Li , L. Dai , Q. Liu , L. Song , J. Wang , Y. Li , J. Tian , B. Ding , et al., ACS Nano 2014, 8, 6633. 24963790 10.1021/nn502058j

[14] J. Liu , L. Song , S. Liu , Q. Jiang , Q. Liu , N. Li , Z.-G. Wang , B. Ding , Nano Lett. 2018, 18, 3328. 29708760 10.1021/acs.nanolett.7b04812

[15] Q. Jiang , C. Song , J. Nangreave , X. Liu , L. Lin , D. Qiu , Z.-G. Wang , G. Zou , X. Liang , H. Yan , et al., J. Am. Chem. Soc. 2012, 134, 13396. 22803823 10.1021/ja304263n

[16] S. Palazzolo , M. Hadla , C. R. Spena , S. Bayda , V. Kumar , F. Lo Re , M. Adeel , I. Caligiuri , F. Romano , G. Corona , et al., ACS Med. Chem. Lett. 2019, 10, 517. 30996789 10.1021/acsmedchemlett.8b00557 PMC6466551

[17] P. D. Halley , C. R. Lucas , E. M. McWilliams , M. J. Webber , R. A. Patton , C. Kural , D. M. Lucas , J. C. Byrd , C. E. Castro , Small 2016, 12, 308. 26583570 10.1002/smll.201502118 PMC4879968

[18a] C. Pérez-Arnaiz , N. Busto , J. M. Leal , B. García , J. Phys. Chem. B 2014, 118, 1288; 24417409 10.1021/jp411429g

[18b] H. Ijäs , B. Shen , A. Heuer-Jungemann , A. Keller , M. A. Kostiainen , T. Liedl , J. A. Ihalainen , V. Linko , bioRxiv 2020, 10.1101/2020.05.13.088054. PMC8034656 33660776

[19] S. A. Abraham , K. Edwards , G. Karlsson , S. MacIntosh , L. D. Mayer , C. McKenzie , M. B. Bally , Biochim. Biophys. Acta Biomembr. 2002, 1565, 41. 10.1016/s0005-2736(02)00507-2 12225851

[20] Z. Fülöp , R. Gref , T. Loftsson , Int. J. Pharm. 2013, 454, 559. 23850794 10.1016/j.ijpharm.2013.06.058

[21] M. Airoldi , G. Barone , G. Gennaro , A. M. Giuliani , M. Giustini , Biochemistry 2014, 53, 2197. 24641674 10.1021/bi401687v

[22] H. L. Miller , S. Contera , A. J. M. Wollman , A. Hirst , K. E. Dunn , S. Schröter , D. O'Connell , M. C. Leake , Nanotechnology 2020, 31, 235605. 32125281 10.1088/1361-6528/ab7a2b

[23] F. Kollmann , S. Ramakrishnan , B. Shen , G. Grundmeier , M. A. Kostiainen , V. Linko , A. Keller , ACS Omega 2018, 3, 9441. 31459078 10.1021/acsomega.8b00934 PMC6644410

[24] C. Möser , J. S. Lorenz , M. Sajfutdinow , D. M. Smith , Int. J. Mol. Sci. 2018, 19, 3482. 10.3390/ijms19113482 PMC6274923 30404153

[25] V. Maximov , V. Reukov , A. A. Vertegel , J. Drug Delivery Sci. Technol. 2009, 19, 311.

[26] A. Jaekel , P. Stegemann , B. Saccà , Molecules 2019, 24, 3694. 10.3390/molecules24203694 PMC6832416 31615123

[27] A. Ora , E. Järvihaavisto , H. Zhang , H. Auvinen , H. A. Santos , M. A. Kostiainen , V. Linko , Chem. Commun. 2016, 52, 14161. 10.1039/c6cc08197e 27869278

[28] J. K. Kiviaho , V. Linko , A. Ora , T. Tiainen , E. Järvihaavisto , J. Mikkilä , H. Tenhu , Nonappa , M. A. Kostiainen , Nanoscale 2016, 8, 11674. 27219684 10.1039/c5nr08355a

[29] V. Linko , M. Eerikäinen , M. A. Kostiainen , Chem. Commun. 2015, 51, 5351. 10.1039/c4cc08472a 25594847

[30] A. Sprengel , P. Lill , P. Stegemann , K. Bravo-Rodriguez , E.-C. Schöneweiß , M. Merdanovic , D. Gudnason , M. Aznauryan , L. Gamrad , S. Barcikowski , et al., Nat. Commun. 2017, 8, 14472. 28205515 10.1038/ncomms14472 PMC5316895

[31] H. Ijäs , I. Hakaste , B. Shen , M. A. Kostiainen , V. Linko , ACS Nano 2019, 13, 5959. 30990664 10.1021/acsnano.9b01857 PMC7076726

[32] G. Grossi , M. D. E. Jepsen , J. Kjems , E. S. Andersen , Nat. Commun. 2017, 8, 992. 29051565 10.1038/s41467-017-01072-8 PMC5648847

[33] S. Juul , F. Iacovelli , M. Falconi , S. L. Kragh , B. Christensen , R. Frøhlich , O. Franch , E. L. Kristoffersen , M. Stougaard , K. W. Leong , et al., ACS Nano 2013, 7, 9724. 24168393 10.1021/nn4030543

[34] Z. Zhao , J. Fu , S. Dhakal , A. Johnson-Buck , M. Liu , T. Zhang , N. W. Woodbury , Y. Liu , N. G. Walter , H. Yan , Nat. Commun. 2016, 7, 10619. 26861509 10.1038/ncomms10619 PMC4749968

[35] S. Zhao , F. Duan , S. Liu , T. Wu , Y. Shang , R. Tian , J. Liu , Z.-G. Wang , Q. Jiang , B. Ding , ACS Appl. Mater. Interfaces 2019, 11, 11112. 30874429 10.1021/acsami.8b21724

[36] V. Linko , S. Nummelin , L. Aarnos , K. Tapio , J. J. Toppari , M. A. Kostiainen , Nanomaterials 2016, 6, 139. 10.3390/nano6080139 PMC5224616 28335267

[37] B. J. H. M. Rosier , A. J. Markvoort , B. Gumí Audenis , J. A. L. Roodhuizen , A. den Hamer , L. Brunsveld , T. F. A. de Greef , Nat. Catal. 2020, 3, 295 32190819 10.1038/s41929-019-0403-7 PMC7080557

[38a] G. Chen , D. Liu , C. He , T. R. Gannett , W. Lin , Y. Weizmann , J. Am. Chem. Soc. 2015, 137, 3844; 25622178 10.1021/ja512665z

[38b] J. J. Fakhoury , C. K. McLaughlin , T. W. Edwardson , J. W. Conway , H. F. Sleiman , Biomacromolecules 2014, 15, 276; 24328173 10.1021/bm401532n

[38c] J.-W. Keum , J.-H. Ahn , H. Bermudez , Small 2011, 7, 3529. 22025353 10.1002/smll.201101804

[39] H. Lee , A. K. R. Lytton-Jean , Y. Chen , K. T. Love , A. I. Park , E. D. Karagiannis , A. Sehgal , W. Querbes , C. S. Zurenko , M. Jayaraman , et al., Nat. Nanotechnol. 2012, 7, 389. 22659608 10.1038/nnano.2012.73 PMC3898745

[40] H. Xue , F. Ding , J. Zhang , Y. Guo , X. Gao , J. Feng , X. Zhu , C. Zhang , Chem. Commun. 2019, 55, 4222. 10.1039/c9cc00175a 30896698

[41] S. M. Douglas , I. Bachelet , G. M. Church , Science 2012, 335, 831. 22344439 10.1126/science.1214081

[42] S. Li , D. Jiang , Z. T. Rosenkrans , T. E. Barnhart , E. B. Ehlerding , D. Ni , J. W. Engle , W. Cai , Nano Lett. 2019, 19, 7334. 31518140 10.1021/acs.nanolett.9b02958 PMC6876547

[43a] J. Li , H. Pei , B. Zhu , L. Liang , M. Wei , Y. He , N. Chen , D. Li , Q. Huang , C. Fan , ACS Nano 2011, 5, 8783; 21988181 10.1021/nn202774x

[43b] L. Zhang , G. Zhu , L. Mei , C. Wu , L. Qiu , C. Cui , Y. Liu , I.-T. Teng , W. Tan , ACS Appl. Mater. Interfaces 2015, 7, 24069; 26440045 10.1021/acsami.5b06987 PMC4898273

[43c] Y. Qu , J. Yang , P. Zhan , S. Liu , K. Zhang , Q. Jiang , C. Li , B. Ding , ACS Appl. Mater. Interfaces 2017, 9, 20324; 28570804 10.1021/acsami.7b05890

[43d] M. Nishikawa , M. Matono , S. Rattanakiat , N. Matsuoka , Y. Takakura , Immunology 2008, 124, 247; 18217956 10.1111/j.1365-2567.2007.02762.x PMC2566629

[43e] S. Rattanakiat , M. Nishikawa , H. Funabashi , D. Luo , Y. Takakura , Biomaterials 2009, 30, 5701; 19604576 10.1016/j.biomaterials.2009.06.053

[43f] K. Mohri , E. Kusuki , S. Ohtsuki , N. Takahashi , M. Endo , K. Hidaka , H. Sugiyama , Y. Takahashi , Y. Takakura , M. Nishikawa , Biomacromolecules 2015, 16, 1095. 25775113 10.1021/bm501731f

[44] V. J. Schüller , S. Heidegger , N. Sandholzer , P. C. Nickels , N. A. Suhartha , S. Endres , C. Bourquin , T. Liedl , ACS Nano 2011, 5, 9696. 22092186 10.1021/nn203161y

[45a] X. Liu , Y. Xu , T. Yu , C. Clifford , Y. Liu , H. Yan , Y. Chang , Nano Lett. 2012, 12, 4254; 22746330 10.1021/nl301877k PMC3808986

[45b] S. Sellner , S. Kocabey , K. Nekolla , F. Krombach , T. Liedl , M. Rehberg , Biomaterials 2015, 53, 453. 25890742 10.1016/j.biomaterials.2015.02.099

[46] D. E. J. G. J. Dolmans , D. Fukumura , R. K. Jain , Nat. Rev. Cancer 2003, 3, 380. 12724736 10.1038/nrc1071

[47] X. Huang , P. K. Jain , I. H. El-Sayed , M. A. El-Sayed , Lasers Med. Sci. 2008, 23, 217. 17674122 10.1007/s10103-007-0470-x

[48a] X. Zhuang , X. Ma , X. Xue , Q. Jiang , L. Song , L. Dai , C. Zhang , S. Jin , K. Yang , B. Ding , et al., ACS Nano 2016, 10, 3486; 26950644 10.1021/acsnano.5b07671 PMC4837698

[48b] K.-R. Kim , D. Bang , D.-R. Ahn , Biomater. Sci. 2016, 4, 605. 26674121 10.1039/c5bm00467e

[49] Q. Jiang , Y. Shi , Q. Zhang , N. Li , P. Zhan , L. Song , L. Dai , J. Tian , Y. Du , Z. Cheng , et al., Small 2015, 11, 5134. 26248642 10.1002/smll.201501266

[50] Y. Du , Q. Jiang , N. Beziere , L. Song , Q. Zhang , D. Peng , C. Chi , X. Yang , H. Guo , G. Diot , et al., Adv. Mater. 2016, 28, 10000. 27679425 10.1002/adma.201601710

[51] Y. Amir , E. Ben-Ishay , D. Levner , S. Ittah , A. Abu-Horowitz , I. Bachelet , Nat. Nanotechnol. 2014, 9, 353. 24705510 10.1038/nnano.2014.58 PMC4012984

[52] S. Li , Q. Jiang , S. Liu , Y. Zhang , Y. Tian , C. Song , J. Wang , Y. Zou , G. J. Anderson , J.-Y. Han , et al., Nat. Biotechnol. 2018, 36, 258. 29431737 10.1038/nbt.4071

[53] J. Liu , L. Song , S. Liu , S. Zhao , Q. Jiang , B. Ding , Angew. Chem. Int. Ed. 2018, 57, 15486; 10.1002/anie.201809452 30288887

[54] L. Song , Q. Jiang , J. Liu , N. Li , Q. Liu , L. Dai , Y. Gao , W. Liu , D. Liu , B. Ding , Nanoscale 2017, 9, 7750. 28581004 10.1039/c7nr02222k

[55] D. Jiang , Z. Ge , H.-J. Im , C. G. England , D. Ni , J. Hou , L. Zhang , C. J. Kutyreff , Y. Yan , Y. Liu , et al., Nat. Biomed. Eng. 2018, 2, 865. 30505626 10.1038/s41551-018-0317-8 PMC6258029

[56] S. Lin , R. I. Gregory , Nat. Rev. Cancer 2015, 15, 321. 25998712 10.1038/nrc3932 PMC4859809

[57] Y. Ke , S. Lindsay , Y. Chang , Y. Liu , H. Yan , Science 2008, 319, 180. 18187649 10.1126/science.1150082

[58] D. Wang , Y. Fu , J. Yan , B. Zhao , B. Dai , J. Chao , H. Liu , D. He , Y. Zhang , C. Fan , et al., Anal. Chem. 2014, 86, 1932. 24447268 10.1021/ac403661z

[59] A. Kuzuya , Y. Sakai , T. Yamazaki , Y. Xu , M. Komiyama , Nat. Commun. 2011, 2, 449. 21863016 10.1038/ncomms1452 PMC3265375

[60] H.-K. Walter , J. Bauer , J. Steinmeyer , A. Kuzuya , C. M. Niemeyer , H.-A. Wagenknecht , Nano Lett. 2017, 17, 2467. 28249387 10.1021/acs.nanolett.7b00159

[61a] K. S. Park , S. W. Shin , M. S. Jang , W. Shin , K. Yang , J. Min , S.-W. Cho , B.-K. Oh , J. W. Bae , S. Jung , et al., Sci. Rep. 2015, 5, 18497; 26678430 10.1038/srep18497 PMC4683441

[61b] W. Zhou , D. Li , C. Xiong , R. Yuan , Y. Xiang , ACS Appl. Mater. Interfaces 2016, 8, 13303. 27195747 10.1021/acsami.6b03165

[62] D. Selnihhin , S. M. Sparvath , S. Preus , V. Birkedal , E. S. Andersen , ACS Nano 2018, 12, 5699. 29763544 10.1021/acsnano.8b01510

[63] C. Zhu , J. Yang , J. Zheng , S. Chen , F. Huang , R. Yang , Anal. Chem. 2019, 91, 15599. 31762260 10.1021/acs.analchem.9b03659

[64a] G. P. Acuna , F. M. Möller , P. Holzmeister , S. Beater , B. Lalkens , P. Tinnefeld , Science 2012, 338, 506; 23112329 10.1126/science.1228638

[64b] A. Puchkova , C. Vietz , E. Pibiri , B. Wünsch , M. Sanz Paz , G. P. Acuna , P. Tinnefeld , Nano Lett. 2015, 15, 8354. 26523768 10.1021/acs.nanolett.5b04045

[65] S. E. Ochmann , C. Vietz , K. Trofymchuk , G. P. Acuna , B. Lalkens , P. Tinnefeld , Anal. Chem. 2017, 89, 13000. 29144729 10.1021/acs.analchem.7b04082

[66a] H. Pei , N. Lu , Y. Wen , S. Song , Y. Liu , H. Yan , C. Fan , Adv. Mater. 2010, 22, 4754; 20839255 10.1002/adma.201002767 PMC3071359

[66b] S. Liu , W. Su , Z. Li , X. Ding , Biosens. Bioelectron. 2015, 71, 57. 25884735 10.1016/j.bios.2015.04.006

[67] A. Abi , M. Lin , H. Pei , C. Fan , E. E. Ferapontova , X. Zuo , ACS Appl. Mater. Interfaces 2014, 6, 8928. 24802004 10.1021/am501823q

[68] S. Han , W. Liu , S. Yang , R. Wang , ACS Omega 2019, 4, 11025. 31460200 10.1021/acsomega.9b01166 PMC6649092

[69] T. Funck , F. Nicoli , A. Kuzyk , T. Liedl , Angew. Chem. Int. Ed. 2018, 57, 13495; 10.1002/anie.201807029 30084527

[70] M. Godonoga , T.-Y. Lin , A. Oshima , K. Sumitomo , M. S. L. Tang , Y.-W. Cheung , A. B. Kinghorn , R. M. Dirkzwager , C. Zhou , A. Kuzuya , et al., Sci. Rep. 2016, 6, 21266. 26891622 10.1038/srep21266 PMC4759581

[71] D. Jiang , Y. Sun , J. Li , Q. Li , M. Lv , B. Zhu , T. Tian , D. Cheng , J. Xia , L. Zhang , et al., ACS Appl. Mater. Interfaces 2016, 8, 4378. 26878704 10.1021/acsami.5b10792

[72] H. Zhang , J. Chao , D. Pan , H. Liu , Y. Qiang , K. Liu , C. Cui , J. Chen , Q. Huang , J. Hu , et al., Nat. Commun. 2017, 8, 14738. 28382928 10.1038/ncomms14738 PMC5384221

[73] K. Liu , D. Pan , Y. Wen , H. Zhang , J. Chao , L. Wang , S. Song , C. Fan , Y. Shi , Small 2018, 14, 1701718. 10.1002/smll.201701718 29283218

[74] Y. Sakai , M. S. Islam , M. Adamiak , S. C.-C. Shiu , J. A. Tanner , J. G. Heddle , Genes 2018, 9, 571. 10.3390/genes9120571 PMC6315403 30477184

[75] S. Rinker , Y. Ke , Y. Liu , R. Chhabra , H. Yan , Nat. Nanotechnol. 2008, 3, 418. 18654566 10.1038/nnano.2008.164 PMC2556356

[76] M. Li , J. Liu , H. Zhao , L. Song , X. Mao , Z. Ge , Q. Li , F. Li , X. Zuo , ACS Appl. Bio Mater. 2020, 3, 53. 10.1021/acsabm.9b00841 35019426

[77] Y. Huang , M.-K. Nguyen , A. K. Natarajan , V. H. Nguyen , A. Kuzyk , ACS Appl. Mater. Interfaces 2018, 10, 44221. 30525378 10.1021/acsami.8b19153

[78] T. Funck , T. Liedl , W. Bae , Appl. Sci. 2019, 9, 3006.

[79] P. S. Kwon , S. Ren , S.-J. Kwon , M. E. Kizer , L. Kuo , M. Xie , D. Zhu , F. Zhou , F. Zhang , D. Kim , et al., Nat. Chem. 2020, 12, 26. 31767992 10.1038/s41557-019-0369-8 PMC6925649

[80] H. Pei , Y. Wan , J. Li , H. Hu , Y. Su , Q. Huang , C. Fan , Chem. Commun. 2011, 47, 6254. 10.1039/c1cc11660f 21541424

[81] L. Yuan , M. Giovanni , J. Xie , C. Fan , D. T. Leong , NPG Asia Mater. 2014, 6, e112.

[82] M. Giovanni , M. I. Setyawati , C. Y. Tay , H. Qian , W. S. Kuan , D. T. Leong , Adv. Funct. Mater. 2015, 25, 3840.

[83] J. Wang , M. C. Leong , E. Z. W. Leong , W. S. Kuan , D. T. Leong , Anal. Chem. 2017, 89, 6900. 28548485 10.1021/acs.analchem.7b01508

[84] D. Jiang , C. G. England , W. Cai , J. Controlled Release 2016, 239, 27. 10.1016/j.jconrel.2016.08.013 PMC5037045 27527555

[85] S. D. Perrault , W. M. Shih , ACS Nano 2014, 8, 5132. 24694301 10.1021/nn5011914 PMC4046785

[86] N. Ponnuswamy , M. M. C. Bastings , B. Nathwani , J. H. Ryu , L. Y. T. Chou , M. Vinther , W. A. Li , F. M. Anastassacos , D. J. Mooney , W. M. Shih , Nat. Commun. 2017, 8, 15654. 28561045 10.1038/ncomms15654 PMC5460023

[87] K.-R. Kim , Y.-D. Lee , T. Lee , B.-S. Kim , S. Kim , D.-R. Ahn , Biomaterials 2013, 34, 5226. 23587443 10.1016/j.biomaterials.2013.03.074

[88] V. Gubala , L. F. Harris , A. J. Ricco , M. X. Tan , D. E. Williams , Anal. Chem. 2012, 84, 487. 22221172 10.1021/ac2030199

[89] J.-W. Keum , H. Bermudez , Chem. Commun. 2009, 7036. 10.1039/b917661f 19904386

[90] Q. Mei , X. Wei , F. Su , Y. Liu , C. Youngbull , R. Johnson , S. Lindsay , H. Yan , D. Meldrum , Nano Lett. 2011, 11, 1477. 21366226 10.1021/nl1040836 PMC3319871

[91] C. E. Castro , F. Kilchherr , D.-N. Kim , E. L. Shiao , T. Wauer , P. Wortmann , M. Bathe , H. Dietz , Nat. Methods 2011, 8, 221. 21358626 10.1038/nmeth.1570

[92] J. Hahn , S. F. J. Wickham , W. M. Shih , S. D. Perrault , ACS Nano 2014, 8, 8765. 25136758 10.1021/nn503513p PMC4174095

[93a] G. Zuccheri , DNA Nanotechnology, Springer, New York, NY, 2018;

[93b] G. Zuccheri , B. Samorì , DNA Nanotechnology, Humana Press, Totowa, NJ, 2011.

[94] C. Kielar , Y. Xin , B. Shen , M. A. Kostiainen , G. Grundmeier , V. Linko , A. Keller , Angew. Chem. Int. Ed. 2018, 57, 9470; 10.1002/anie.201802890 29799663

[95a] T. G. Martin , H. Dietz , Nat. Commun. 2012, 3, 1103; 23033079 10.1038/ncomms2095 PMC3493638

[95b] S. Ramakrishnan , G. Krainer , G. Grundmeier , M. Schlierf , A. Keller , Small 2017, 13, 1702100; 10.1002/smll.201702100 29024433

[95c] D. Wang , Z. Da , B. Zhang , M. A. Isbell , Y. Dong , X. Zhou , H. Liu , J. Y. Y. Heng , Z. Yang , RSC Adv. 2015, 5, 58734.

[96] J. A. L. Roodhuizen , P. J. T. M. Hendrikx , P. A. J. Hilbers , T. F. A. de Greef , A. J. Markvoort , ACS Nano 2019, 13, 10798. 31502824 10.1021/acsnano.9b05650 PMC6764110

[97] V. Linko , B. Shen , K. Tapio , J. J. Toppari , M. A. Kostiainen , S. Tuukkanen , Sci. Rep. 2015, 5, 15634. 26492833 10.1038/srep15634 PMC4616047

[98a] K. E. Bujold , A. Lacroix , H. F. Sleiman , Chem 2018, 4, 495;

[98b] J. R. Burns , S. Howorka , Nanomaterials 2019, 9, 490. 10.3390/nano9040490 PMC6523550 30934927

[99] Y. Hu , Z. Chen , Z. Hou , M. Li , B. Ma , X. Luo , X. Xue , Molecules 2019, 24, 2091. 10.3390/molecules24112091 PMC6600315 31159358

[100] S. Naskar , M. Gosika , H. Joshi , P. K. Maiti , J. Phys. Chem. C 2019, 123, 9461.

[101] S. Kocabey , H. Meinl , I. S. MacPherson , V. Cassinelli , A. Manetto , S. Rothenfusser , T. Liedl , F. S. Lichtenegger , Nanomaterials 2014, 5, 47. 28346998 10.3390/nano5010047 PMC5312849

[102] K. Kishi , T. Yasuda , Y. Ikehara , K. Sawazaki , W. Sato , R. Iida , Am. J. Hum. Genet. 1990, 47, 121. 2349940 PMC1683738

[103] Q. Y. Liu , M. Ribecco , S. Pandey , P. R. Walker , M. Sikorska , Ann. N. Y. Acad. Sci. 1999, 887, 60. 10668464 10.1111/j.1749-6632.1999.tb07922.x

[104] S. Goltry , N. Hallstrom , T. Clark , W. Kuang , J. Lee , C. Jorcyk , W. B. Knowlton , B. Yurke , W. L. Hughes , E. Graugnard , Nanoscale 2015, 7, 10382. 25959862 10.1039/c5nr02283e PMC4457601

[105] A. Lacroix , E. Vengut-Climent , D. de Rochambeau , H. F. Sleiman , ACS Cent. Sci. 2019, 5, 882. 31139724 10.1021/acscentsci.9b00174 PMC6535766

[106] H. Auvinen , H. Zhang , Nonappa , A. Kopilow , E. H. Niemelä , S. Nummelin , A. Correia , H. A. Santos , V. Linko , M. A. Kostiainen , Adv. Healthcare Mater. 2017, 6, 1700692. 10.1002/adhm.201700692 28738444

[107] S. Ramakrishnan , B. Shen , M. A. Kostiainen , G. Grundmeier , A. Keller , V. Linko , ChemBioChem 2019, 20, 2818. 31163091 10.1002/cbic.201900369

[108] S. Surana , A. R. Shenoy , Y. Krishnan , Nat. Nanotechnol. 2015, 10, 741. 26329110 10.1038/nnano.2015.180 PMC4862568

[109] K. Xia , H. Kong , Y. Cui , N. Ren , Q. Li , J. Ma , R. Cui , Y. Zhang , J. Shi , Q. Li , et al., ACS Appl. Mater. Interfaces 2018, 10, 15442. 29668248 10.1021/acsami.8b02626

[110] D. Mathur , I. L. Medintz , Adv. Healthcare Mater. 2019, 8, 1801546. 10.1002/adhm.201801546 PMC9285959 30843670

[111] M. M. C. Bastings , F. M. Anastassacos , N. Ponnuswamy , F. G. Leifer , G. Cuneo , C. Lin , D. E. Ingber , J. H. Ryu , W. M. Shih , Nano Lett. 2018, 18, 3557. 29756442 10.1021/acs.nanolett.8b00660

[112] K.-R. Kim , S. J. Kang , A.-Y. Lee , D. Hwang , M. Park , H. Park , S. Kim , K. Hur , H. S. Chung , C. Mao , et al., Biomaterials 2019, 195, 1. 30593871 10.1016/j.biomaterials.2018.12.026

[113] K.-R. Kim , T. Lee , B.-S. Kim , D.-R. Ahn , Chem. Sci. 2014, 5, 1533.

[114] A. Stopar , L. Coral , S. Di Giacomo , A. F. Adedeji , M. Castronovo , Nucleic Acids Res. 2018, 46, 995. 29216375 10.1093/nar/gkx1204 PMC5778535

[115] A. Suma , A. Stopar , A. W. Nicholson , M. Castronovo , V. Carnevale , Nucleic Acids Res. 2020, 48, 4672. 32043111 10.1093/nar/gkaa080 PMC7229852

[116] Q. Liu , G. Liu , T. Wang , J. Fu , R. Li , L. Song , Z.-G. Wang , B. Ding , F. Chen , ChemPhysChem 2017, 18, 2977. 28856771 10.1002/cphc.201700809

[117] Y. Kim , P. Yin , Angew. Chem. Int. Ed. 2020, 59, 700; 10.1002/anie.201911664 PMC6940523 31595637

[118] D. H. Schaffert , A. H. Okholm , R. S. Sørensen , J. S. Nielsen , T. Tørring , C. B. Rosen , A. L. B. Kodal , M. R. Mortensen , K. V. Gothelf , J. Kjems , Small 2016, 12, 2634. 27032044 10.1002/smll.201503934

[119] Y. Li , R. Schulman , Nano Lett. 2019, 19, 3751. 31140279 10.1021/acs.nanolett.9b00888

[120] A. Lacroix , T. G. W. Edwardson , M. A. Hancock , M. D. Dore , H. F. Sleiman , J. Am. Chem. Soc. 2017, 139, 7355. 28475327 10.1021/jacs.7b02917

[121] N. P. Agarwal , M. Matthies , F. N. Gür , K. Osada , T. L. Schmidt , Angew. Chem. Int. Ed. 2017, 56, 5460; 10.1002/anie.201608873 28295864

[122] J. Mikkilä , A.-P. Eskelinen , E. H. Niemelä , V. Linko , M. J. Frilander , P. Törmä , M. A. Kostiainen , Nano Lett. 2014, 14, 2196. 24627955 10.1021/nl500677j

[123] N. A. Estrich , A. Hernandez-Garcia , R. de Vries , T. H. LaBean , ACS Nano 2017, 11, 831. 28048935 10.1021/acsnano.6b07291

[124] Y. Ahmadi , E. de Llano , I. Barišić , Nanoscale 2018, 10, 7494. 29637957 10.1039/c7nr09461b

[125] F. M. Anastassacos , Z. Zhao , Y. Zeng , W. M. Shih , J. Am. Chem. Soc. 2020, 142, 3311. 32011869 10.1021/jacs.9b11698

[126] I. Kopatz , R. Zalk , Y. Levi-Kalisman , E. Zlotkin-Rivkin , G. A. Frank , S. Kler , Nanoscale 2019, 11, 10160. 30994643 10.1039/c8nr10113b

[127] X. Xu , S. Fang , Y. Zhuang , S. Wu , Q. Pan , L. Li , X. Wang , X. Sun , B. Liu , Y. Wu , Materials 2019, 12, 949. 10.3390/ma12060949 PMC6470866 30901888

[128] A. Hernandez-Garcia , N. A. Estrich , M. W. T. Werten , J. R. C. van der Maarel , T. H. LaBean , F. A. de Wolf , M. A. Cohen Stuart , R. de Vries , ACS Nano 2017, 11, 144. 27936577 10.1021/acsnano.6b05938

[129] P. O′Neill , P. W. K. Rothemund , A. Kumar , D. K. Fygenson , Nano Lett. 2006, 6, 1379. 16834415 10.1021/nl0603505

[130] S. Ramakrishnan , L. Schärfen , K. Hunold , S. Fricke , G. Grundmeier , M. Schlierf , A. Keller , G. Krainer , Nanoscale 2019, 11, 16270. 31455950 10.1039/c9nr04460d

[131] G. D. Hamblin , K. M. M. Carneiro , J. F. Fakhoury , K. E. Bujold , H. F. Sleiman , J. Am. Chem. Soc. 2012, 134, 2888. 22283197 10.1021/ja2107492

[132] V. Cassinelli , B. Oberleitner , J. Sobotta , P. Nickels , G. Grossi , S. Kempter , T. Frischmuth , T. Liedl , A. Manetto , Angew. Chem. Int. Ed. 2015, 54, 7795; 10.1002/anie.201500561 25980669

[133] M. Kalinowski , R. Haug , H. Said , S. Piasecka , M. Kramer , C. Richert , ChemBioChem 2016, 17, 1150. 27225865 10.1002/cbic.201600061

[134] S. Raniolo , S. Croce , R. P. Thomsen , A. H. Okholm , V. Unida , F. Iacovelli , A. Manetto , J. Kjems , A. Desideri , S. Biocca , Nanoscale 2019, 11, 10808. 31134260 10.1039/c9nr02006c

[135] T. Gerling , M. Kube , B. Kick , H. Dietz , Sci. Adv. 2018, 4, eaau1157. 30128357 10.1126/sciadv.aau1157 PMC6097813

[136] F. A. S. Engelhardt , F. Praetorius , C. H. Wachauf , G. Brüggenthies , F. Kohler , B. Kick , K. L. Kadletz , P. N. Pham , K. L. Behler , T. Gerling , et al., ACS Nano 2019, 13, 5015. 30990672 10.1021/acsnano.9b01025 PMC6992424

[137] A. Rajendran , M. Endo , Y. Katsuda , K. Hidaka , H. Sugiyama , J. Am. Chem. Soc. 2011, 133, 14488. 21859143 10.1021/ja204546h

[138] T. Gerling , H. Dietz , Angew. Chem. Int. Ed. 2019, 58, 2680; 10.1002/anie.201812463 PMC6984961 30694591

[139] J. W. Conway , C. K. McLaughlin , K. J. Castor , H. Sleiman , Chem. Commun. 2013, 49, 1172. 10.1039/c2cc37556g 23287884

[140a] C. Kielar , Y. Xin , X. Xu , S. Zhu , N. Gorin , G. Grundmeier , C. Möser , D. M. Smith , A. Keller , Molecules 2019, 24, 2577; 10.3390/molecules24142577 PMC6680526 31315177

[140b] B. Zhu , Y. Zhao , J. Dai , J. Wang , S. Xing , L. Guo , N. Chen , X. Qu , L. Li , J. Shen , et al., ACS Appl. Mater. Interfaces 2017, 9, 18434; 28547989 10.1021/acsami.7b04784

[140c] Y. Xin , C. Kielar , S. Zhu , C. Sikeler , X. Xu , C. Möser , G. Grundmeier , T. Liedl , A. Heuer-Jungemann , D. M. Smith , et al., Small 2020, 16, 1905959. 10.1002/smll.201905959 32130783

[141] F. Praetorius , B. Kick , K. L. Behler , M. N. Honemann , D. Weuster-Botz , H. Dietz , Nature 2017, 552, 84. 29219963 10.1038/nature24650

[142] J. Geigert , The Challenge of CMC Regulatory Compliance for Biopharmaceuticals and Other Biologics, Springer, New York, NY, 2013.

[143] C. A. Stein , D. Castanotto , Mol. Ther. 2017, 25, 1069. 28366767 10.1016/j.ymthe.2017.03.023 PMC5417833

[144a] P. Jitawatanarat , T. L. O'Connor , E. B. Kossoff , E. G. Levine , K. Chittawatanarat , N. Ngamphaiboon , J. Breast Cancer 2014, 17, 356; 25548584 10.4048/jbc.2014.17.4.356 PMC4278055

[144b] Union for International Cancer Control, *2014 Review of Cancer Medicines on the WHO List of Essential Medicines*, World Health Organization, Geneva, **2014**.

